# Transformation of organic micropollutants along hyporheic flow in bedforms of river-simulating flumes

**DOI:** 10.1038/s41598-021-91519-2

**Published:** 2021-06-22

**Authors:** Anna Jaeger, Malte Posselt, Jonas L. Schaper, Andrea Betterle, Cyrus Rutere, Claudia Coll, Jonas Mechelke, Muhammad Raza, Karin Meinikmann, Andrea Portmann, Phillip J. Blaen, Marcus A. Horn, Stefan Krause, Jörg Lewandowski

**Affiliations:** 1 Department Ecohydrology, Leibniz Institute of Freshwater Ecology and Inland Fisheries, Berlin, Germany; 2 Geography Department, Humboldt University Berlin, Berlin, Germany; 3 Department of Environmental Science, Stockholm University, Stockholm, Sweden; 4 Center for Applied Geoscience, Eberhard Karls University of Tübingen, Tübingen, Germany; 5 Department of Civil, Environmental and Mechanical Engineering, University of Trento, Trento, Italy; 6 Department of Ecological Microbiology, University of Bayreuth, Bayreuth, Germany; 7 Eawag, Swiss Federal Institute of Aquatic Science and Technology, Dübendorf, Switzerland; 8 Institute of Biogeochemistry and Pollutant Dynamics, ETH Zürich, Zürich, Switzerland; 9 Institute of Applied Geosciences, Technical University of Darmstadt, Darmstadt, Germany; 10 IWW Water Centre, Mülheim an der Ruhr, Germany; 11 Julius Kühn Institute – Federal Research Centre for Cultivated Plants, Institute for Ecological Chemistry, Plant Analysis and Stored Product Protection, Berlin, Germany; 12 Civil and Environmental Engineering, Colorado School of Mines, Golden, CO USA; 13 School of Geography, Earth and Environmental Sciences, University of Birmingham, Birmingham, UK; 14 Yorkshire Water, Leeds, UK; 15 Institute of Microbiology, Leibniz University of Hannover, Hannover, Germany; 16 Université Claude Bernard Lyon 1, Ecologie des Hydrosystèmes Naturels et Anthropisés (LEHNA), Villeurbanne, France

**Keywords:** Biogeochemistry, Environmental sciences, Hydrology

## Abstract

Urban streams receive increasing loads of organic micropollutants from treated wastewaters. A comprehensive understanding of the in-stream fate of micropollutants is thus of high interest for water quality management. Bedforms induce pumping effects considerably contributing to whole stream hyporheic exchange and are hotspots of biogeochemical turnover processes. However, little is known about the transformation of micropollutants in such structures. In the present study, we set up recirculating flumes to examine the transformation of a set of micropollutants along single flowpaths in two triangular bedforms. We sampled porewater from four locations in the bedforms over 78 days and analysed the resulting concentration curves using the results of a hydrodynamic model in combination with a reactive transport model accounting for advection, dispersion, first-order removal and retardation. The four porewater sampling locations were positioned on individual flowpaths with median solute travel times ranging from 11.5 to 43.3 h as shown in a hydrodynamic model previously. Highest stability was estimated for hydrochlorothiazide on all flowpaths. Lowest detectable half-lives were estimated for sotalol (0.7 h) and sitagliptin (0.2 h) along the shortest flowpath. Also, venlafaxine, acesulfame, bezafibrate, irbesartan, valsartan, ibuprofen and naproxen displayed lower half-lives at shorter flowpaths in the first bedform. However, the behavior of many compounds in the second bedform deviated from expectations, where particularly transformation products, e.g. valsartan acid, showed high concentrations. Flowpath-specific behavior as observed for metformin or flume-specific behavior as observed for metoprolol acid, for instance, was attributed to potential small-scale or flume-scale heterogeneity of microbial community compositions, respectively. The results of the study indicate that the shallow hyporheic flow field and the small-scale heterogeneity of the microbial community are major controlling factors for the transformation of relevant micropollutants in river sediments.

## Introduction

Hyporheic zones are areas of streambeds where surface water (SW) permeates the sediment. The process of SW infiltration into the hyporheic zone, causing subsurface flow of streambed porewater (PW) and potential exfiltration to the SW is termed hyporheic exchange flow^1,2^. Hyporheic exchange flows of various scales create hotspots of biogeochemical activity rendering the hyporheic zone “the liver” of a river^3^. The high reactivity derives from dynamic geochemical conditions induced by the water flow combined with a high surface area of the sediment particles providing room for the development of diverse microbial communities^1,4^. The transport of oxygen, nutrients and bioavailable organic carbon to the subsurface sediment generates a spatially and temporally heterogeneous ecohydrological environment. The resulting steep redox gradients and the enhanced biological activity along hyporheic flowpaths are responsible for the transformative function of hyporheic zones in particular and enable various metabolic and co-metabolic transformation processes^4–7^. For this reason, hyporheic zones have been studied as hotspots for turnover and removal of nutrients, fine particulate matter and more recently also emerging contaminants, such as organic micropollutants.

Concerns have been raised over the impacts of micropollutants on ecosystem health and drinking water production, particularly in urban areas. Many urban streams are heavily modified and their streambeds are partly sealed. Moreover, they receive high amounts of treated wastewater containing a range of synthetic organic compounds, such as pharmaceuticals or personal care products^8,9^. Hence, there is a growing interest in identifying stream characteristics affecting the micropollutant removal capacity of urban streams. In contemporary urban planning there is a trend towards river rehabilitation and the various benefits of less engineered rivers are increasingly recognised. In particular, the significance of a conductive streambed for water quality and aquatic habitats in urban stream management plans is more and more acknowledged^10,11^. Micropollutant removal in sediment–water interfaces has been investigated in a number of recent studies, in particular with regards to bank-filtration scenarios^12–14^ and along vertical flowpaths in losing streams^15,16^. Transformation processes of many micropollutants in sediments have been found to be promoted in such transition regions due to their specific redox-sensitivity and their dependency on the diversity of the microbial activities and communities involved^15,17^. Despite the significance of bedforms for streambed hyporheic exchange^18–21^ very few studies have analyzed the transformation of micropollutants along hyporheic flowpaths through streambed structures. Peter et al.^22^ induced artificial hyporheic exchange in a creek and found water quality improvement along the hyporheic flowpaths with more than 50% removal of 78% of the analysed compounds on longer hyporheic flowpaths. Li et al.^23^ investigated attenuation of micropollutants in bedforms built in recirculating flumes, similar to the setup of the present study. They found formation of four transformation products (TPs) exclusively in the sediment (carbamazepine-10,11-epoxide, saluamine, metoprolol acid, and 1-naphthol).

Hyporheic exchange has been systematically investigated by simplifying streambed geometries to triangular bedforms^24–27^. They contribute to overall reach-scale hyporheic exchange as individual bedforms and as bedform-sequences^18,28^. Bedforms induce hyporheic exchange by the so-called “pumping-effect”. High water pressure building up on the stoss side of the bedform contrasts low pressure on the leeside of the triangle forcing the water through the bedform^20^. Hyporheic travel times and flowpath distributions depend on sediment properties such as hydraulic conductivity, the SW flow velocity and the inclination of the bedform itself^24,26,29^. The diverse flowpaths within the hyporheic zone of bedforms induce a variety of biogeochemical conditions creating reactive hotspots of microbial activities and redox reactions on small scales^27,30,31^. The “purification” caused by centimeter-scale hyporheic exchange has been shown to considerably influence the stream water quality. Short flowpaths in shallow hyporheic zones are expected to be of particular relevance for turnover processes, as supply of substrate and nutrients is continuous, the shallow microbial community is potentially very active and steep redox gradients occur in the shallow sediment^32–34^. Hence, the identification of micropollutant transformation processes caused by shallow hyporheic exchange in bedforms is important to understand and eventually predict reach-scale attenuation of micropollutants and formation of TPs.

The present study was conducted as part of an interdisciplinary joint experiment comprised of several subprojects. In previous studies we described the experiment, in which in total 20 river-simulating flumes were set up to investigate the influence of bacterial diversity and hyporheic exchange on the fate of organic micropollutants in the SW. The previous studies addressed the complete setup and functioning of the experimental design^35^, the fate of micropollutants and the individual influence of the treatments in the SW^36,37^, and a hydrodynamic model estimating hyporheic exchange in the flumes^38^. The present study focuses on the transformation of micropollutants in the hyporheic zone, i.e. in the PW, of two specific flumes on a centimeter-scale. The flumes were set up as replicates and are here referred to as Flume 1 and Flume 2. Both contained triangular bedforms equipped with PW samplers. Supported by hydrodynamic and reactive transport models, the study aims to investigate the behavior and fate of a set of organic micropollutants and related TPs along specific shallow hyporheic flowpaths in two of the bedforms of each flume. Specifically, the objectives were to identify (1) the effect of specific flowpath characteristics, such as residence times, on degradation of parent compounds and (2) the conditions favoring formation of TPs.

## Methods

### Experimental setup

**Figure 1:**
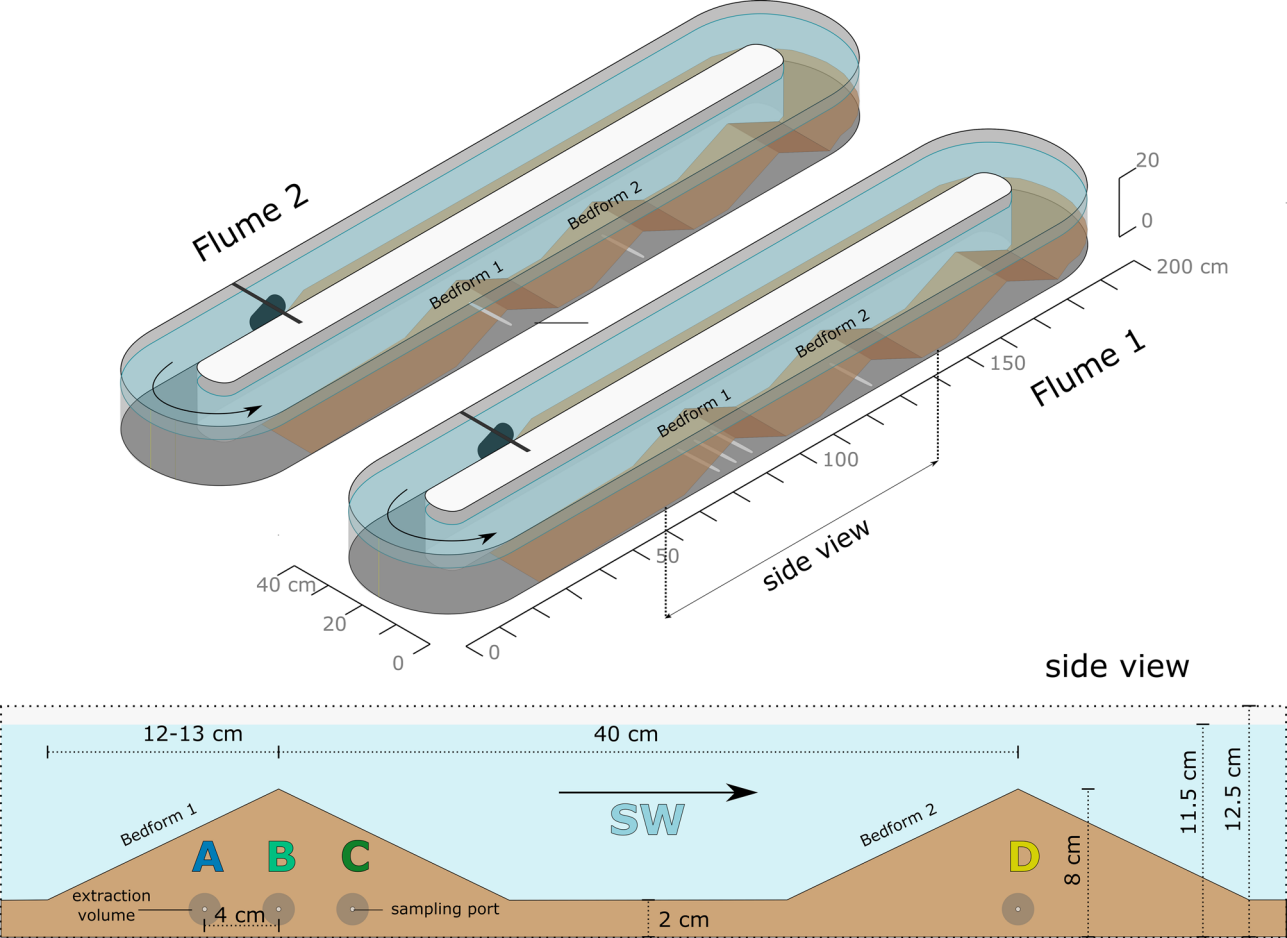
Schematic of Flumes 1 and 2. Flume 1 held porewater samplers in positions A, B, C and D. Flume 2 held porewater samplers in positions B and D. The measures represent ideal conditions obtained after bedform formation. Figure adapted from Jaeger et al.^35^.

The project deployed circular flumes of 2 m length (Fig. 1) at the EcoLab facilities of the University of Birmingham, UK. The flumes were placed inside a white, well-ventilated tent covering the setup from direct solar radiation and rain. They were leveled, filled with 20 L of a sediment mixture consisting of oven dried (120 °C for 24 h) and washed commercial sand mixed with sediment from the wastewater-impacted River Erpe in Berlin, Germany, (Table 1) and 60 L of deionized water. Aquarium pumps were installed and a nutrient mix (Supplementary Table S1) was added to the SW. The pumps were set to induce a SW flow of 8 cm s^−1^. The bacterial communities in the flumes were left for a pre-incubation period of 12 days. On day 9 of the pre-incubation period, the bedforms were created using custom-made wooden plates. In both, Flume 1 and Flume 2, three bedforms of 8 cm height were formed with 40 cm distance between their tips in the channel opposite of the pump. The sediment surface on the pump side of the flumes was left flat. At the same time, PW samplers (Standard Rhizons, Rhizosphere Research Products B.V., Wageningen, The Netherlands) were installed into the sediment at 1.5 cm above the bottom of the flume (Fig. 1). In Flume 1, four samplers were installed, three of them (A, B, C) in Bedform 1 in 4 cm distance and one (D) in Bedform 2 resembling position B in Bedform 1. In Flume 2, two samplers were deployed, at positions resembling B in Bedform 1 and D in Bedform 2 of Flume 1 (Fig. 1). All flumes of the project contained one PW sampler in position D^36^. The reason for installing 3 samplers in a row in Bedform 1 of Flume 1 was to be able to follow different flowpaths within a single bedform. We added an additional sampler in position B of Flume 1 to examine the general difference in flowpaths and reactive transport between Bedform 1 and Bedform 2.

**Table 1:** Boundary conditions of Flumes 1 and 2, as well as shared sediment properties (mean ± sd).

	Flume 1	Flume 2	Days of measurement
Surface water flow velocity at the bedform side of the flume [cm s^−1^]	7.0 ± 1.8	7.2 ± 0.3	27, 47, 82
Drop of bedform heights by day 27 [%]	4	5	27
Drop of bedform heights by day 82 [%]	32	33	82
Water level [cm]	11.3 ± 0.2	11.4 ± 0.3	27, 47, 82
pH	8.3	8.3	− 4, 45
O_2_ in the surface water [%]	101.8 ± 5.9	103.1 ± 5.9	28, 36, 44, 82
Sediment composition	2.13 kg sand + 2 L Erpe sediment		Measurements performed with initial sediment mixtures
K_f_ at 10 °C [m s^−1^]	3.14 × 10^−4^ ± 4%		
Porosity [%]	35		
Total carbon [%]	0.01		
Fine gravel (2–6.3 mm) [%]	5		
Coarse sand (0.63–2 mm) [%]	6		
Medium sand (0.2–0.63 mm) [%]	82		
Fine sand (0.063–0.2 mm) [%]	6		
< 0.063 mm [%]	< 1		

The first day after pre-incubation (day 0), SW and PW samples were taken and right after a set of 31 synthetic micropollutants (Table S1 in ref.^35^) dissolved in methanol was injected into the SW of each flume. The flumes ran for the following 78 days and SW and PW were sampled 10 more times (days 1, 2, 3, 7, 14, 21, 28, 42, 56 and 78). The increasing sampling time intervals were chosen, because we expected the highest change of concentrations in the SW and the occurrence of breakthrough curves in the PW within the first days after injection, while we assumed little change within the last weeks of the experiment. Approximately 10 ml of PW were extracted from the Rhizons using PE syringes. Considering the 10 cm length of the Rhizon and the sediment porosity (Table 1), a PW sample intake from within a radius of roughly 0.95 cm around the samplers was assumed (Fig. 1). Additional nutrient mixes were added to the SW at days 10 and 46 (Supplementary Table S1). Upon evaporation of SW, the flumes were refilled with 3 to 5 L deionized water 6 times. A description of the specific sediment properties and boundary conditions in Flumes 1 and 2 is shown in Table 1. A detailed description of the main project’s experimental design including the timeline, the list of all injected compounds and background conditions can be found in Jaeger et al.^35^, which describes the overall experimental setup for the investigation of the fate of micropollutants in the SW.

### Chemical and bacterial analyses

Aliquots of SW and PW samples were immediately stored at − 20 °C, and analysed for micropollutants at Stockholm University, Sweden, using direct injection reversed-phase ultrahigh-performance liquid chromatography electrospray ionization triple quadrupole tandem mass spectrometry according to a method presented in Posselt et al.^39^. For details on QA/QC applied within the overall experiment, see Posselt et al.^36^. Values below limit of quantification (LOQ) were replaced by LOQ·2^−0.5^ (Supplementary Table S2).

A second set of aliquots of samples taken at days 0, 21, 42 and 78 was analysed at Birmingham University, UK, for concentrations of NO_3_^−^, NO_2_^–^, NH_4_^+^, PO_4_^3−^, total nitrogen (TN) and dissolved organic carbon (DOC). Samples were stored at − 20 °C and SW samples were filtered through 0.45 μm nylon filters (Thames Restek, UK) prior to analysis. Due to the Rhizon sampler pore size of 0.15 μm, PW samples did not require additional filtering. Concentrations of NO_3_^−^, NO_2_^−^, NH_4_^+^, PO_4_^3−^ were determined using a Skalar (Breda, Netherlands) SAN +  + continuous flow analyzer and concentrations of DOC and TN were determined using a Shimadzu (Kyoto, 126 Japan) TOC-L analyzer^35^. PW dissolved oxygen profiles of Bedform 1 and 2 of Flume 2 were recorded at day 1 using oxygen needle sensors (Unisense A/S, Aarhus, Denmark) attached to an aluminum pole (0.5 cm diameter) which was height-adjusted using a manual micromanipulator.

Sediment samples were taken from the flat sediment sections of each flume at days 0, 21 and 56, stored at − 80 °C and shipped on dry ice to the University of Bayreuth, Germany, for the analysis of the bacterial community structure. DNA extraction was performed following the rapid method for extraction of total nucleic acids from environmental samples^40^. After removal of co-extracted RNA, DNA concentration was measured with Quant-iT PicoGreen DNA assay kit following manufacturer’s protocol (Invitrogen, Germany) and the Tecan Infinite plate reader (Tecan, Switzerland). Subsequently, the gene copy numbers of bacterial 16S rRNA genes were quantified by quantitative PCR^36^. Sequencing of the 16S rRNA amplicons was performed using the Illumina Miseq amplicon sequencing platform. Operational taxonomic units defined at 97% similarity were used to determine bacterial taxa and to calculate bacterial diversity indices following Posselt et al.^36^ and Rutere et al.^41^. The copy numbers of 16S rRNA genes per gram of dry sediment for Flume 1 (day 0: 1.29*10^6^; day 21: 0.00; day 56: 2.62*10^7^) and Flume 2 (day 0: 2.17*10^6^; day 21: 3.25*10^6^; day 56: 1.33*10^7^) indicated, that the flumes had developed a bacterial community of similar biomass after pre-incubation. The naught value of copy numbers in Flume 1 at day 21 was regarded an instrumental outlier due to the high values at days 0 and 56.

### Hydrodynamic model

The hyporheic flow field feeding the respective PW samplers was simulated by a particle backtracking model as described in Betterle et al.^38^. Simulations included a fully coupled 2D description of the joint surface and hyporheic flow, combining the Navier–Stokes equations for the surface flow and the Brinkman equations for the hyporheic flow. In a second phase, a specifically-developed inverse tracking algorithm was adopted to backtrack single flowpaths. At each sampler position, 10,000 particles (conservative compounds) were seeded in the model according to a bivariate normal distribution of a horizontal variance $\sigma_{{\text{x}}}^{2}$ = 5 mm^2^ and a vertical variance of $\sigma_{{\text{x}}}^{2}$ = 2.5 mm^2^ around the sampling location and tracked back to their likely origin at the sediment-surface water interface. As described in Betterle et al.^38^, simulations identified the trajectories of water particles and provided an estimate of the probability distribution of flowpath lengths and travel times expected to be sampled at the four sampling locations. The results of the model were used to illustrate and compare the trajectories of the different flowpaths in the bedforms. In addition, estimated distributions of both flowpath lengths and resulting advective PW velocities were subsequently used as prior probability density functions during parameter inference in the reactive transport model.

### Reactive transport model

Similar to previous work^15^, the one-dimensional advection–dispersion transport equation was used to simulate the reactive transport along the four Flowpaths a, b, c, and d in Flume 1 for all parent compounds displaying more than 5% of samples above LOQ. The transport equation can be written as:

1 $$R\frac{\partial c}{\partial t}={D}_{h}\frac{{\partial }^{2}c}{{\partial x}^{2}}-v\frac{\partial c}{\partial x}-kc$$

where *R* is the retardation coefficient (–), *c* is the concentration of a compound (µg L^−1^) at time *t* (h), *D*_*h*_ (m^2^ h^−1^) denotes the effective hydrodynamic dispersion coefficient, *v* (m h^−1^) the PW velocity along the specific flowpath, and *k* (h^−1^) is the first-order removal rate constant. The model was run independently for each flowpath because the hydrodynamic model demonstrated that Samplers A, B and C were not positioned on the same streamline^38^. Thus, for all four flowpaths, SW concentrations were set as time-varying upper boundary conditions. The SW concentrations of day 0 were set to 11.5 µg L^−1^, which corresponds to the calculated initial concentration of all injected compounds after being mixed with the SW volume. A Neuman (2nd type) boundary condition was set to zero at a distance of 0.25 m for all flowpaths. For all compounds the measured concentration break through curves of the first 21 days of the experiment were used for parameter inference. A simulation period of 21 days was chosen because for the majority of parent compounds the breakthrough had occurred and changes in measured concentration at the sampling locations after day 21 were relatively small or steady, respectively (Supplementary Fig. S1). Limiting the model to 21 days minimized the computational demand. Moreover, considerable changes in morphology and SW velocities occurred after day 21 (Table 1), and thus the assumption of steady state transport implied in Eq. (1) was no longer justified.

The Bayesian parameter optimization algorithm DREAM was implemented for parameter inference^42,43^. Using 20 evolutionary Monte Carlo Markov chains, the algorithm estimates posterior probability density distributions (posteriors) of all model parameters. The flowpath length and the PW velocity *v* were provided as Gaussian prior probability density distributions (priors) for each DREAM run as calculated by the hydrodynamic model for each flowpath. Posteriors of *D*_*h*_ estimated from transport simulations of hydrochlorothiazide, were used as Gaussian priors during the simulation of all other compounds for each respective flowpath. We followed this approach because hydrochlorothiazide showed the most conservative behavior of all parent compounds investigated in the present study. The compound-specific parameters *k* and *R* were estimated individually for each flowpath and each compound using uniform priors (*k*: 1*10^−5^ to 4 h^−1^, *R*: 1 to 49). The posterior values of estimated *k* were converted to half-lives (DT50s). We calculated compound specific DT50 thresholds (Supplementary Table S4). For each compound we considered DT50s corresponding to a reduction in concentration by two times the measurement precision (rel. SD) as thresholds on each flow path (Supplementary Table S4). Median DT50 values exceeding the thresholds were set to “inf”, as they represent degradation that was not measurable with the present methods.

## Results

### Concentration time trends of parent compounds and TPs in the bedforms

40 compounds were analysed in SW and PW samples of Flumes 1 and 2, with 19 of the compounds representing TPs (Supplementary Table S2). For 28 of the measured compounds, more than 5% of all samples showed concentrations above LOQ (Supplementary Fig. S1). Parent compounds with concentrations below LOQ in more than 95% of all samples were metoprolol, propranolol and tramadol (for atenolol only measurements of samplers B were available which were all below LOQ). They were instantly degraded in Flumes 1 and 2 due to the relatively high bacterial diversity in the sediment^36^. The parent compounds acesulfame, bezafibrate, furosemide, ibuprofen, irbesartan, ketoprofen, metformin, naproxen, sitagliptin, sotalol and valsartan were degraded in both SW and PW within the first 30 days. The parent compounds 1H-benzotriazole, carbamazepine, clofibric acid, diclofenac, gemfibrozil, hydrochlorothiazide, sulfamethoxazole and venlafaxine showed lower degradability in either SW or PW and some of them were still measurable at day 78 of the experiment (Supplementary Fig. S1). TPs that did not yield concentrations above LOQ in more than 5% of all samples were either not formed or readily degraded after formation and were thus not measurable. Of the remaining TPs, 1-methyl-1H-benzotriazole, 2/4-chlorobenzoic acid and metoprolol acid were formed and subsequently almost completely removed within the first 20 days in SW and/or PW, while chlorothiazide, carbamazepine-10,11-epoxide and valsartan acid showed rising trends in the SW and some of the PW samplers (Supplementary Fig. S1). Finally, O-desmethylvenlafaxine, 10,11-dihydroxy carbamazepine and 4-hydroxydiclofenac exhibited formation-degradation patterns without clear trends (Supplementary Fig. S1). Due to the solute transport time within the bedform, the first 14 days of the experiment were most interesting for the present study, which is why this time period will be the focus of the discussion.

**Figure 2:**
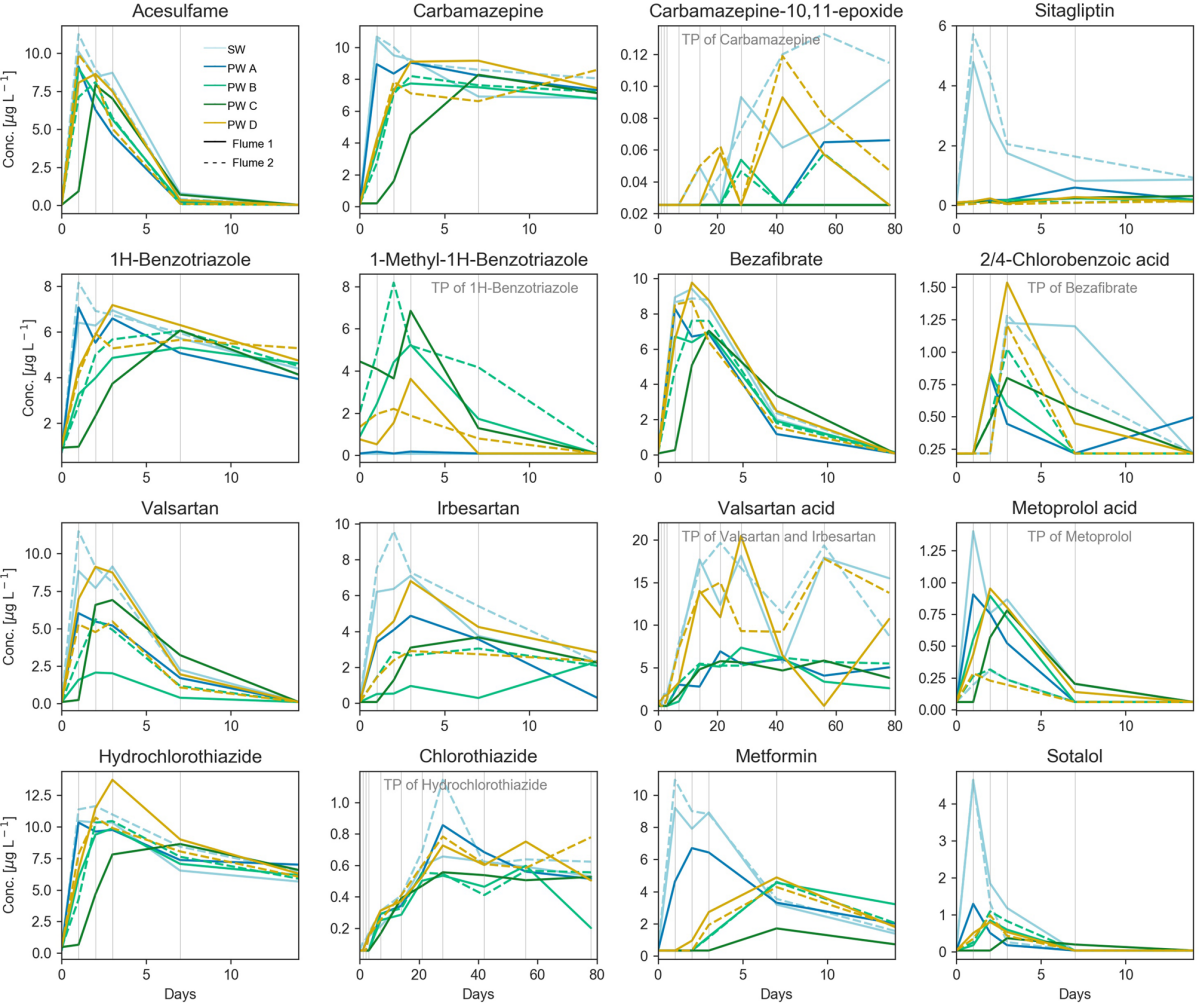
Measured concentrations in the SW, in PW Samplers A, B, C and D in Flume 1 and PW Samplers B and D in Flume 2 of selected compounds and related TPs. Grey vertical lines indicate sampling days. Note the differences in scales of the x- and y-axes. For concentrations of all compounds see Supplementary Fig. S1 (78 days) and Fig. S2 (7 days).

The behavior of compounds along the different flowpaths varied between compounds. For some parent compounds the concentrations rose in succession of the sampler positions. In these cases, concentration peaks arrived first at Sampler A, then Samplers B and D simultaneously, and finally Sampler C, for example 1H-benzotriazole, carbamazepine, metformin and hydrochlorothiazide. In other cases, the behavior was more complex, e.g. valsartan and irbesartan, where the behavior between Samplers B and D and additionally in Flumes 1 and 2 differed considerably. The differences in sampler positions B and D were particularly visible in the formation patterns of valsartan acid, carbamazepine-10,11-epoxide and also partly chlorothiazide. Interestingly, for metoprolol acid the major difference occurred between flumes as opposed to bedforms, as the positions B and D behaved similarly in the single flumes (Fig. 2). Generally, an increase in concentration of TPs is attributed to biological formation processes. However, when investigating concentration dynamics of TPs, it is important to consider that higher concentrations not necessarily imply generally higher formation. TPs themselves may often further degrade or display different sorption properties than their parent compound^44^. Their total concentrations are often a result of simultaneous formation and dissipation dynamics. Consequently, the concentration differences between samplers are caused by differences in formation to dissipation ratios rather than formation alone. For simplicity, in the present work, the term net-formation refers to formation-to-dissipation ratio, i.e. “higher net-formation” implies that either formation is higher or dissipation is lower along the flowpath to one sampler compared to another.

### Oxygen and nutrient dynamics

The PW dissolved oxygen concentration profiles measured at day 1 in Flume 2 indicate that oxygen was readily consumed in the sediment (Supplementary Fig. S4). Within a few millimeters, the oxygen concentrations dropped below detectability. In Bedform 2 a slight difference between up- and downstream side of the bedform might indicate advective transport of oxygen into the bedform with the infiltrating SW. However, the prevailing conditions in both bedforms were clearly anoxic. Although the measurements were conducted only once, it can be assumed that the oxygen distribution remained similar throughout the experiment as the hyporheic exchange rather decreased over time due to reduced flow velocities (Table 1), likely further limiting the oxygen supply from the SW to the sediment.

**Figure 3:**
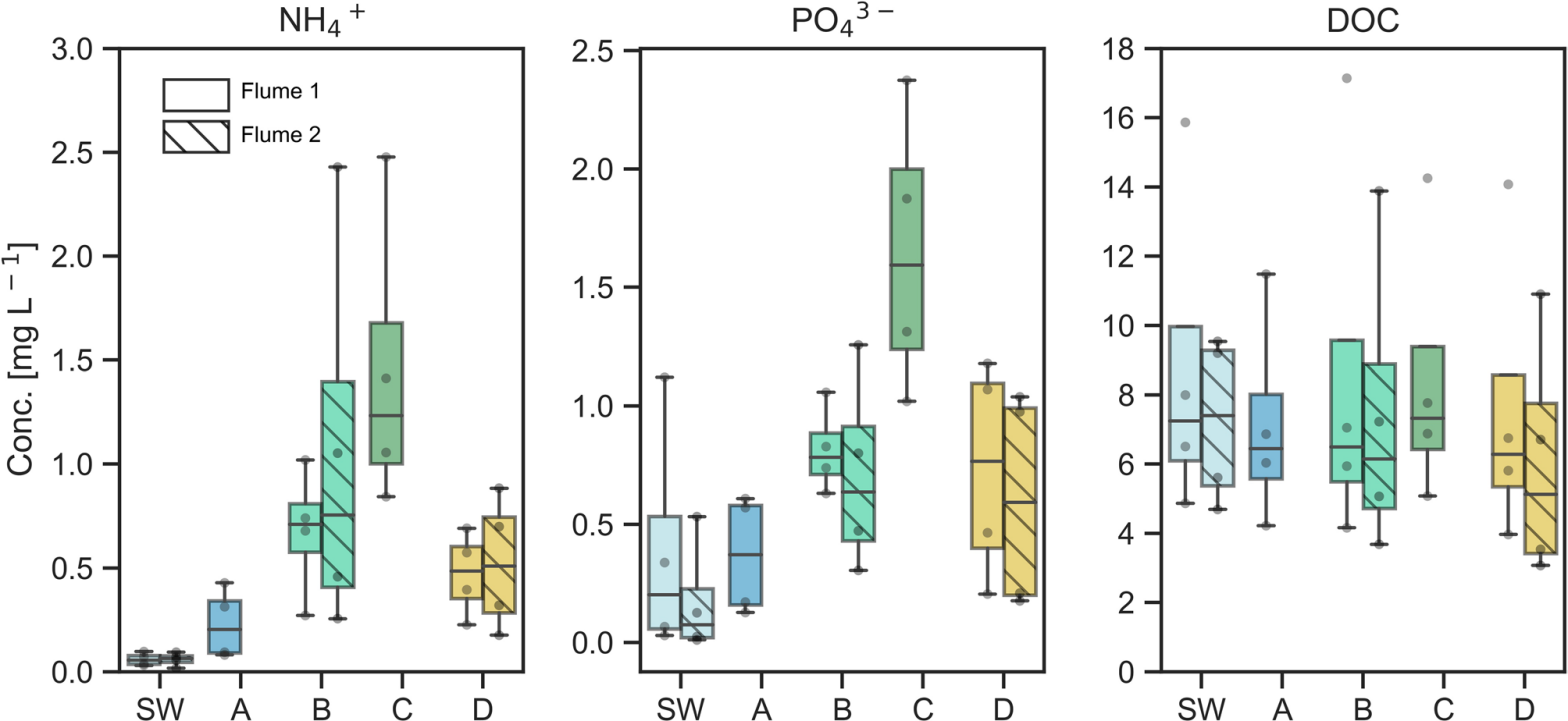
Boxplots of concentrations of NH_4_^+^, PO_4_^3−^ and DOC in the SW, in Bedform 1 (Samplers A, B, and C) and Bedform 2 (Sampler D) of Flumes 1 and 2 aggregated over the PW sampling days 0, 21, 42 and 78 (n = 4).

The NH_4_^+^ and PO_4_^3−^ concentrations rising in the succession of Samplers A to C in Flume 1 indicate a gradient of redox conditions along the flowpaths (Fig. 3). Low NH_4_^+^ levels in positions A compared to C can be a result of both nitrification in the zone of higher oxygen availability or assimilation along the flowpaths due to higher microbial activity. Those processes are expected to occur in a relation of 40%:60%^45^. As nitrification and denitrification are often closely coupled ^45^ NO_3_^−^ and NO_2_^−^ produced by nitrification in the small aerobic area prior to Sampler A are apparently readily consumed by denitrifiers, as NO_3_^−^ and NO_2_^−^ concentrations are < LOQ at all three positions. Assimilation of NO_3_^−^ as a cause for the NO_3_^−^ depletion is unlikely as NO_3_^−^ microbial assimilation is suppressed in presence of NH_4_^+^^46^. It was shown previously that residence times in the hyporheic zone determine the fate of nitrogen. At longer residence times net denitrification is prevailing due to decreasing redox potential along the flowpath^47^. This means, while the net nitrification and thus oxic zone caused by SW infiltration does not reach Sampler A, the sampler is likely positioned within a zone of higher redox potential and thus higher nitrification potential than Samplers B and C. The reason, that NH_4_^+^ is not depleted by nitrification is apparently a constant NH_4_^+^ supply by ammonification in the sediment, also confirmed by the generally higher concentrations in the PW compared to the SW.

The increase in PO_4_^3−^ concentration from A to C (Fig. 3) was also caused by redox zonation. PO_4_^3−^ is sorbed to sediment Mn and Fe oxides under high redox potential and is released to the PW by reductive dissolution of Fe^3+^ to Fe^2+^ and Mn^4+^ to Mn^2+^^48^. Hence, Sampler C was likely positioned in a zone of Fe-reducing redox potential. Similar to NH_4_^+^, PW concentrations generally exceeded SW concentrations, indicating that mineralization rate was higher than microbial assimilation rate. Despite the gradient in nitrification caused by redox zonation, the concentrations of DOC did not change considerably along the flowpath. Generally, the differences between Flume 1 and 2 in the median nutrient concentrations in the SW as well as Samplers B and D were small compared to the differences between the median concentrations in the different Samplers A, B and C. This indicates that biogeochemical conditions in the bedforms did not generally differ between flumes of the same sediment mixture. Also median concentrations in Samplers B and D were within the same range as opposed to Samplers A and C (Fig. 3).

### Microbial communities

**Figure 4:**
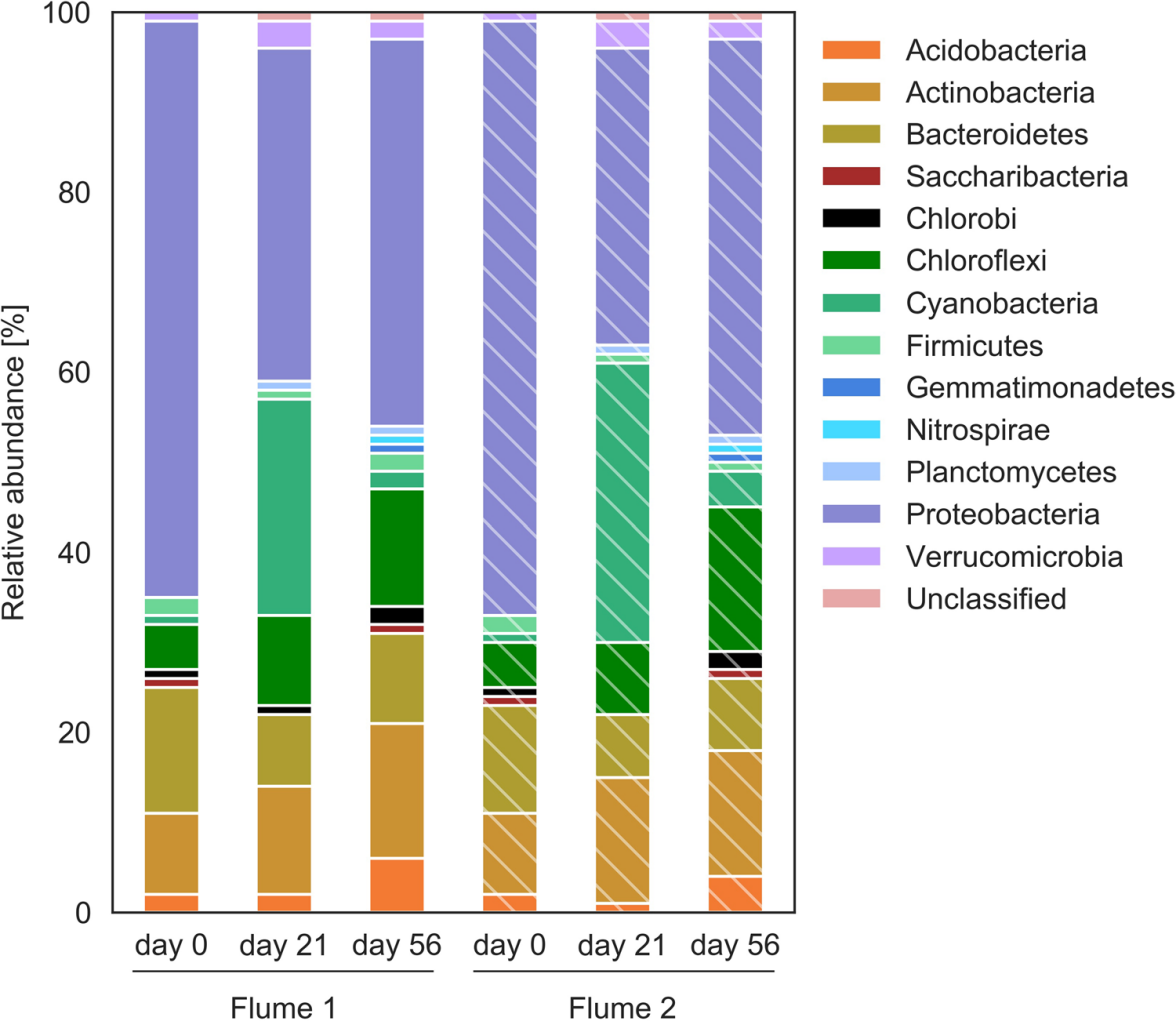
Bacterial community composition at the phylum level in relative abundance values (%) in Flumes 1 and 2 sampled at days 0, 21 and 56, respectively. Samples were taken from the flat sediment area in the flumes.

Differences in relative abundance of phyla between Flume 1 and Flume 2 were marginal (< 5%; Fig. 4). The highest difference was observed in the abundance of cyanobacteria, which was 7% higher on day 21 in Flume 2 than in Flume 1. In both flumes the cyanobacteria increased from 1% to more than 20% (Flume 1: 24%; Flume 2: 31%) of the total community from day 0 to day 21, but declined again to less than 5% by day 56 (Fig. 4).

The overall richness, which is an indicator for the number of species, was relatively balanced over time and between Flume 1 (day 0: 2667; day 21: 2412; day 56: 3609) and Flume 2 (day 0: 2824; day 21: 2107; day 56: 3616). The evenness indicated the distribution of sequences per species increased over time and was slightly higher in Flume 1 (day 0: 0.082; day 21: 0.153; day 56: 0.235) than in Flume 2 (day 0: 0.085; day 21: 0.115; day 56: 0.233). A similar trend was observed for the Shannon diversity index calculated by a combination of richness and evenness in Flume 1 (day 0: 5.39; day 21: 5.91; day 56: 6.74) and Flume 2 (day 0: 5.48; day 21: 5.49; day 56: 6.69). Thus, microbiome analyses suggested that microbial communities were similar in both flumes.

### Hydrodynamic model

**Figure 5:**
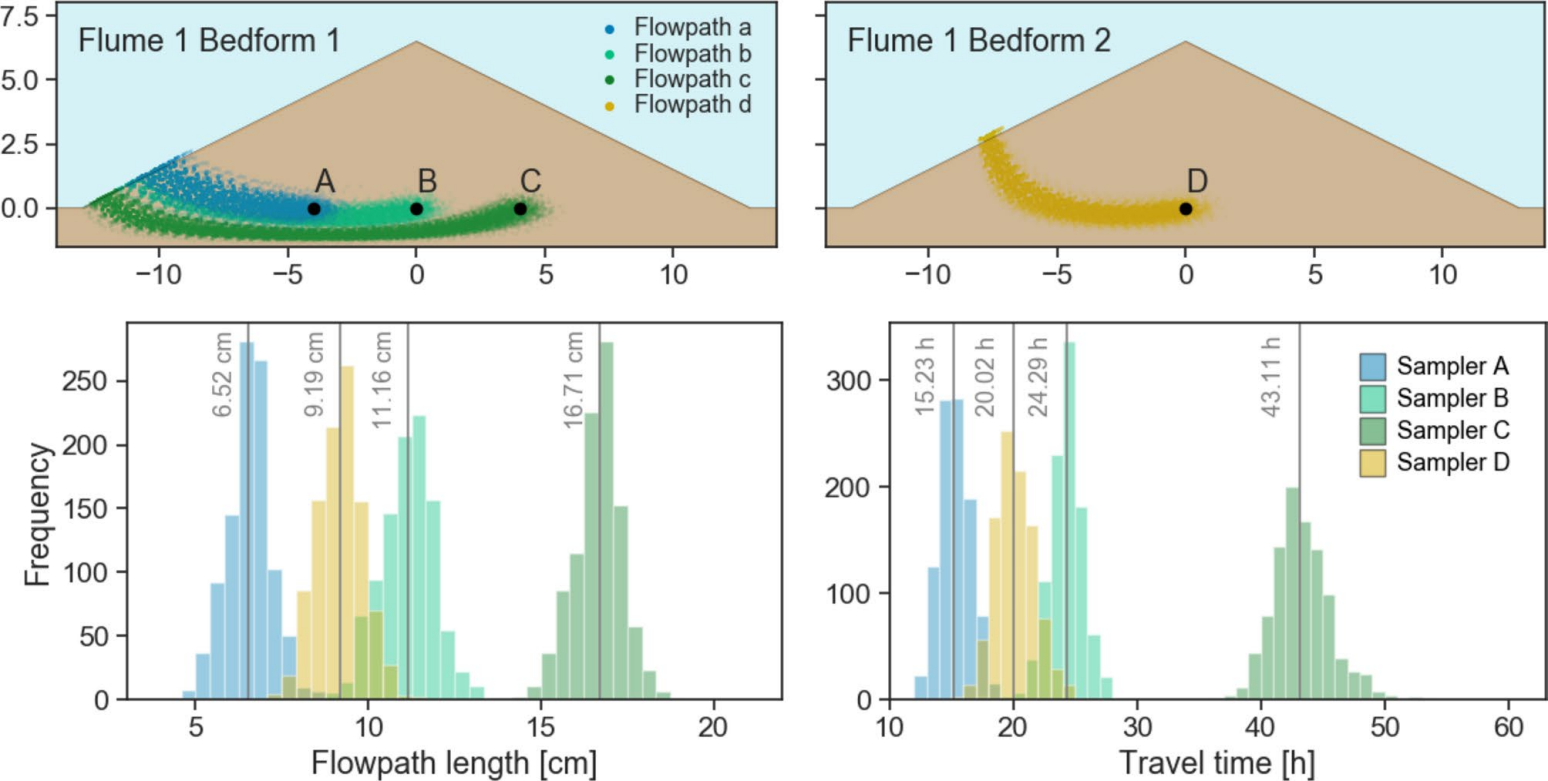
Top: Trajectories of particles along Flowpaths a, b, c and d in Bedforms 1 and 2 calculated by the hydrodynamic model. Bottom: Distribution and median values of flowpath lengths and travel times from SW to PW Samplers A, B, C and D. Figure adapted from Betterle et al.^38^.

The results of the hydrodynamic model (as previously described by Betterle et al.^38^) highlighted that the flowpaths leading to Samplers A and B tended to overlap (i.e. they laid on the same streamline; Fig. 5)^38^. Sampler C, on the other hand, mainly received particles entering the sediment at a lower position on the stoss side and traveling to a large extent below the flowpaths towards Samplers A and B. The median flowpath length and travel times increased from A (5 cm, 11.5 h) to C (16.6 cm, 43.3 h). Conservative solutes arriving at Sampler D, placed in Bedform 2 at the same position as Sampler B in Bedform 1 had a slightly shorter flowpath (9.2 cm) and arrived slightly earlier (20.1 h) than solutes sampled by Sampler B (11.1 cm, 24.3 h)^38^. Therefore, for compounds exhibiting low retardation, the initial sampling interval (24 h) might have been insufficient to capture the different arrival times between Samplers A and B or between Samplers B and D. Note that in the following, flowpaths from the SW towards Samplers A, B, C and D are referred to as Flowpaths a, b, c and d, respectively.

### Reactive transport model

**Figure 6:**
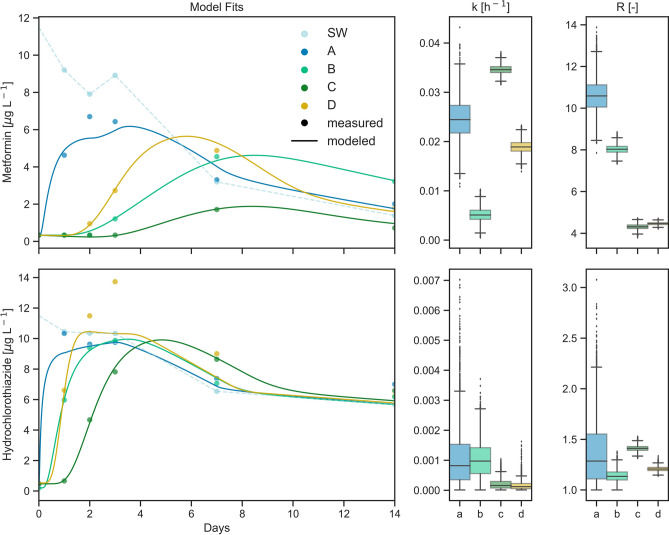
Measured concentrations and modeled breakthrough curves of metformin and hydrochlorothiazide in Flume 1. To the right, the estimated posterior distributions (n = 40,040) of the degradation rate constant *k* and the retardation coefficient *R* are given for each Flowpath a, b, c and d. For model results of all analysed compounds see Supplementary Figs. S5, S6, S7 and S8.

For all parent compounds the estimation of the reactive transport parameters *k* and *R* along Flowpaths a, b, c and d of Flume 1 resulted in model convergence (Table 2, Supplementary Table S3). Root mean square errors (RMSE) were calculated as an indicator for the goodness of fit (Supplementary Table S4), according to which sotalol (0.07), sitagliptin (0.11), gemfibrozil (0.28) and metformin (0.34; Fig. 6) resulted in the best fits and furosemide (2.5) ibuprofen (1.7) and ketoprofen (1.6) obtained the poorest fits on average (Table 2). Poor fits might result from single concentration outliers (e.g. for venlafaxine on Flowpath a or furosemide), but might also generally be caused by model assumptions, such as a time-invariant *k* which might not accurately describe behavior of all compounds. The dispersion coefficients *D*_*h*_ were estimated in a pre-run, fitting the concentration curves of hydrochlorothiazide, which was the most conservative (*k* ≤ 0.001 h^−1^ along a, b, c and d) of all compounds (Fig. 6). The medians and respective interquartile ranges of the posterior distributions of *D*_*h*_ were 0.0015 (0.0008) m^2^ h^−1^ on Flowpath a, 1.1*10^−4^ (5.1*10^−5^) m^2^ h^−1^ on Flowpath b, 5.3*10^−5^ (6.7*10^−6^) m^2^ h^−1^ on Flowpath c and 2.4*10^−5^ (1.4*10^−5^) m^2^ h^−1^ on Flowpath d. Highest median *R* values were estimated for Flowpath a (2.63), while median *R* estimates for b, c and d were similar and remained between 1.17 and 1.34. Highest median *k* values were also found for Flowpath a (0.024 h^−1^) and decreased further from b (0.0156 h^−1^) over c (0.0093 h^−1^) to d (0.0081 h^−1^). However, the trends between flowpaths turned out to be very compound-specific. For better comparability with literature, *k* values were converted to DT50s.

**Table 2:** Medians of parameter estimates for half-lives (DT50s) and retardation coefficients *R* of selected parent compounds and respective inter quartile ranges (in brackets) on Flowpaths a, b, c and d of Flume 1, as well as average root mean square errors (RMSE).

Compound	Retardation coefficient R [–]				Half-life DT50 [h]				RMSE
	a	b	c	d	a	b	c	d	average
1H-Benzotriazole	1.76 (1.14)	1.91 (0.22)	1.90 (0.11)	1.41 (0.09)	53.5 (39.5)	97.3 (42.8)	287 (208)	Inf	0.464
Acesulfame	1.10 (0.13)	1.00 (0.00)	1.00 (0.00)	1.00 (0.01)	6.63 (0.58)	36.6 (2.45)	54.0 (3.34)	54.4 (5.65)	1.498
Bezafibrate	1.09 (0.14)	1.01 (0.02)	1.28 (0.03)	1.05 (0.05)	7.43 (1.05)	36.9 (4.29)	87.5 (13.7)	92.4 (25.9)	0.962
Carbamazepine	2.63 (1.31)	1.61 (0.11)	1.98 (0.06)	1.39 (0.07)	49.0 (39.4	106 (39.2)	284.6(196)	85.1 (26.6)	0.839
Clofibric acid	2.53 (0.84)	1.02 (0.02)	1.17 (0.02)	1.23 (0.04)	Inf	Inf	Inf	Inf	0.904
Diclofenac	5.05 (1.11)	1.39 (0.09)	1.11 (0.03)	1.17 (0.05)	Inf	44.3 (5.15)	139 (29.9)	142 (53.0)	0.913
Furosemide	7.58 (9.09)	1.17 (0.28)	1.42 (0.16)	1.26 (0.16)	Inf	37.0 (10.8)	163 (125)	Inf	2.547
Gemfibrozil	2.62 (0.61)	1.12 (0.07)	1.35 (0.03)	1.31 (0.03)	Inf	154 (39.8)	273 (76.6)	196 (71.3)	0.278
Hydrochlorothiazide	1.29 (0.44)	1.13 (0.08)	1.41 (0.04)	1.21 (0.03)	Inf	Inf	Inf	Inf	0.733
Ibuprofen	5.62 (5.04)	1.20 (0.24)	1.07 (0.11)	1.11 (0.13)	3.71 (1.31)	24.7 (6.12)	53.6 (15.8)	58.3 (31.0)	1.728
Irbesartan	1.53 (0.83)	2.42 (0.23)	1.91 (0.04)	1.38 (0.03)	3.25 (0.32)	5.06 (0.20)	93.1 (10.7)	75.6 (11.9)	0.819
Ketoprofen	1.30 (0.42)	1.03 (0.04)	1.30 (0.05)	1.04 (0.06)	21.1 (7.91)	Inf	63.5 (10.6)	75.7 (26.5)	1.603
Metformin	10.6 (1.07)	8.03 (0.28)	4.31 (0.17)	4.46 (0.09)	28.3 (6.56)	136 (50.2)	20.0 (0.71)	36.7 (3.39)	0.340
Naproxen	4.76 (1.05)	1.05 (0.06)	1.03 (0.04)	1.01 (0.02)	30.1 (12.9)	65.4 (12.9)	67.7 (8.24)	Inf	1.358
Sitagliptin	5.25 (11.6)	11.8 (4.62)	7.42 (0.99)	12.8 (29.0)	0.19 (0.02)	4.99 (0.76)	13.9 (2.13)	5.86 (1.72)	0.113
Sotalol	8.09 (4.88)	3.48 (0.28)	2.83 (0.28)	1.89 (0.10)	0.67 (0.10)	6.89 (0.41)	12.0 (1.55)	6.43 (0.24)	0.072
Sulfamethxazole	3.78 (1.79)	1.10 (0.12)	1.28 (0.05)	1.22 (0.09)	Inf	56.3 (13.7)	29.1 (2.15)	Inf	0.606
Valsartan	2.26 (0.84)	1.16 (0.14)	1.23 (0.03)	1.01 (0.01)	6.37 (0.83)	7.51 (0.27)	74.2 (8.46)	39.7 (4.38)	1.028
Venlafaxine	47.6 (2.10)	12.4 (2.71)	9.23 (1.58)	12.9 (1.34)	0.97 (0.12)	3.59 (0.33)	8.17 (1.35)	4.79 (0.32)	0.620

DT50s exceeding the thresholds (Supplementary Table S4) were set to infinity (inf). For respective values of degradation rate constants *k*, see Supplementary Table S3.

Overall, the fastest degradation was observed for sotalol (DT50: 0.7 to 12 h), sitagliptin (DT50: 0.2 to 14 h) and venlafaxine (DT50: 1.0 to 8.1 h), while degradation of clofibric acid and hydrochlorothiazide was slowest with DT50s above infinity thresholds along all flowpaths. Retardation also varied between compounds and flowpaths. The highest values were estimated for venlafaxine (47.6 to 9.23), metformin (10.6 along a) and sitagliptin (11.8 along b and 12.8 along d). Lowest retardation was estimated for acesulfame, for which *R* was close to 1 on all flowpaths meaning that acesulfame was generally not retarded in the sediment. The fitted breakthrough curves along with the measured values and the estimated distributions of *k* and *R* for all compounds are shown in Supplementary Figs. S5, S6, S7 and S8.

## Discussion

### Transferability to river conditions

The concept of mesocosm experiments implies that experimental conditions are closer to natural conditions than batch experiments. At the same time, conditions are more controlled than in in-situ studies. However, it is also inherent to mesocosm studies that they differ from natural situations in some aspects. In the present study, one major feature deviating from natural conditions is the recirculation. In contrast to rivers, the same parcel of water continuously flows over and through the same sediment in the flumes. Therefore, the flume water is gradually depleted in micropollutant and nutrient concentrations. In-situ the mixture of river and effluent water continuously supplies the sediment with similarly high concentrations, particularly close to the confluence. However, the concentrations decrease along the course of the river. To some degree the spatial decrease in rivers is simulated in flumes by the temporal decrease of micropollutants and nutrients during the experiment.

The decreasing concentrations of the compounds of interest in flumes are useful to study their degradation in SW^35^. Similarly, the changing concentration cause breakthrough curves in the bedforms that can be used to model their degradation. Even in field experiments, changing or fluctuating concentrations are required to estimate reactive transport by fitting a model to concentration curves^15^.

The decreasing supply of micropollutants to the sediment might have affected the microbial community by decreasing toxicity stress or adaptation of the community to changes in the water composition. Other factors, such as the transport of sediment, the pre-incubation phase or more importantly the dilution of the original river sediment with sand have likely affected the community composition even more^36^. A comparison of the bacterial community composition in the flumes and in the original sediment of River Erpe has shown, for instance, that there has been a considerable relative increase in Proteobacter in the flume sediments^49^.

However, maybe most importantly the decrease in nutrients (such as PO_4_^2−^ or NH_4_^+^) and available carbon (DOC) within the study period as opposed to a constant supply in natural rivers has affected the experiment. It might not only have changed the community composition, but also its activity. We can assume that under constant high supply of carbon and nutrients, the microbial activity and therefore the turnover of micropollutants would have been higher^50^. However, there are studies that found higher turnover of micropollutants under oligotrophic conditions, when the microbial community was adapted to the micropollutants as a substrate^51^. Despite of all these deviations of the microbial community in the flumes compared to the stream, flume experiments are the only systematic approach to study in parallel different influencing factors in a less complex system than a natural river. Hence, comparisons to field experiments, especially those from River Erpe, are generally valid when the differing conditions are considered.

Furthermore, some boundary conditions, such as the missing link to the underlying aquifer or the low water depth differ considerably from the situation in many natural rivers. The hydrodynamic circumstances in rivers are very complex and the flow field is affected by various factors, such as the slope, the discharge, the sediment morphology and the connection to the groundwater^52^. A flume study by Fox et al.^21^ has shown that neutral conditions, as opposed to gaining or losing conditions, caused the highest local hyporheic exchange flux. Hence, the present study conditions are closest to a stream of neutral groundwater flux conditions. However, the present study sets a spotlight on shallow flowpaths on a centimeter scale that may occur in various situations and does not aim to represent a certain type of river^21^. For this purpose, the differences in hydrodynamic boundary conditions are of limited relevance.

### Hyporheic flow in the bedforms

The progression of compound breakthrough curves from Samplers A to B to C in the first days of the experiment (Fig. 2) and the rising NH_4_^+^ and PO_4_^3−^ concentrations in the successive sampling positions (Fig. 3) confirm the findings of the hydrodynamic model (Fig. 5) that hyporheic exchange generally occurred in the bedforms^38^. Accordingly, SW entered the sediment at the stoss side of the bedform, traveled through the bedform and re-entered the SW at the leeside within a few days. Although oxygen was consumed prior to PW arrival at Sampler A (Supplementary Fig. S4), the differences in NH_4_^+^ and PO_4_^3−^ concentrations throughout the bedform show that solutes were exposed to an environment varying in redox conditions and microbial activity during passage through the bedform. The bedforms gradually flattened and SW velocities decreased within the 78 days of the experiment causing a decrease in hyporheic exchange over time (Table 1). However, the differences in biogeochemical conditions inside the bedform remained relatively stable, as seen in the high differences in NH_4_^+^ and PO_4_^3−^ between Samplers A, B and C (Fig. 3). Also, for the breakthrough curves in the bedforms and degradation of parent compounds, the first 14 days after injection proved to be most relevant. In this timespan, the hydrodynamic conditions governing the hyporheic flow plausibly remained close to setup conditions.

### Differing conditions along flowpaths a, b, c, and d and between flumes

Solutes sampled at position A were likely the ones exposed to most oxic conditions per travel time considering the short flowpath, the small oxic layer on the stoss side and the low NH_4_^+^ and PO_4_^3−^ concentrations at A. As Flowpaths a and b overlapped to a large extent (Fig. 5), solutes having traveled on from A to B were exposed to more reductive conditions on the way. The flowpath to C laid beneath the other paths, hence solutes traveling to C might have been exposed to a set of conditions differing considerably from Flowpaths a and b. Overall the average conditions on the Flowpath c were likely most reductive considering the high NH_4_^+^ and PO_4_^3−^ concentrations in position C and the long travel times. But were the conditions specific for Bedform 1? In general, the findings from Samplers D and B in both flumes show that observed biogeochemical conditions in different bedforms were relatively similar. While in the hydodynamic model the two flowpaths are more similar to each other than to the other flowpaths, the predicted travel times to D (20 h) were shorter than to B (24 h; Fig. 5). The difference derives from the positioning of the bedforms. Bedform 1 is exposed to relatively direct and undisturbed flow on its stoss side, Bedform 2 is exposed to turbulences caused by Bedform 1^38^.

The differing hydrodynamic flow field in the bedforms likely caused differences in the redox zonation along the flowpaths and might be reflected in slightly lower median values of NH_4_^+^ and PO_4_^3−^ in D than in B (Fig. 3). In addition, the oxygen profile shows a slightly larger oxic layer on the stoss side of Bedform 2 compared to Bedform 1 (Supplementary Fig. S4), also potentially caused by the differing flow fields. Hence, conditions on Flowpath d might have been more reductive than a, but slightly less reductive than b. So, in the succession of a, d, b, c, solutes were likely exposed to a decreasing redox potential. Biogeochemical conditions of Flumes 1 and 2 were similar, which makes them suitable replicates. In the following, the behaviour of all groups of parent compounds and related TPs are discussed. Additionally acesulfame, metformin and sitagliptin are addressed due to special degradation or retardation characteristics observed.

### Hydrochlorothiazide and chlorothiazide

The antidiuretic hydrochlorothiazide is known to undergo primarily abiotic transformation and photolysis^23,53,54^. This behavior was confirmed in the flumes, where hydrochlorothiazide exhibited DT50s of 22.3 and 20.3 days in the SW of Flumes 1 and 2, respectively, but no DT50 below infinity thresholds in the PW yielding the lowest degradation constants k of all modeled compounds (Supplementary Table S3). Similar to River Erpe^39^, concentrations in the PW of the flumes remained very close to SW concentrations after breakthrough (Supplementary Fig. S1).

Chlorothiazide was previously reported to mainly originate from abiotic hydrolysis and photodegradation of hydrochlorothiazide^44^. Concentrations of the TP were initially higher in the SW and Samplers D than in Samplers A, B and C, although hydrochlorothiazide instantly arrived at Sampler A at day 1 and simultaneously at Samplers B and D at day 2 (Supplementary Fig. S2). Concentrations increased in all samplers and the SW until day 20 and then leveled out at around 0.6 µg L^−1^ (Supplementary Fig. S1). However, concentrations in SW, Samplers D and A remained generally higher than concentrations in Samplers B and C indicating that net-formation was higher on shorter flowpaths. As chlorothiazide is an intermediate TP of hydrochlorothiazide degrading further to 4-amino-6-chloro-1,3-benzenedisulfonamide, this transformation step might appear at longer flowpaths featuring higher residence times^55^. In contrast to these findings, chlorothiazide was higher in PW than SW of River Erpe^39^ potentially attributable to higher degradation capacity of the compound in the flume sediments.

### Carbamazepine and TPs carbamazepine-10,11-epoxide and 10,11-dihydroxy carbamazepine

Carbamazepine was the most stable of all injected compounds in the SW with DT50s of 97.5 days in Flume 1 and 81.7 days in Flume 2^36^. Despite its relative persistence, carbamazepine was degraded in the flumes within the timeframe of the experiment and significantly affected by the microbial diversity of the sediment^35^. In the PW, the DT50s ranged from 49 to 285 h. The values were rising from a to d to b to c. Similarly, in the sediment of River Erpe, carbamazepine only degraded in the uppermost 10 cm (9.8 ± 2 h) and was persistent in the deeper layers^15^. The findings indicate that despite its broadly reported persistence (e.g. ref^17,23^), carbamazepine is more likely biodegraded on short, oxic flowpaths. Sorption tests showed that the cationic compound did not sorb irreversibly to the flume sediments^35^. Accordingly, its retardation coefficient was low with 1.4 to 2.6 in the flume sediment, which was slightly lower than in the river sediment (3.6)^15^.

The presence of two TPs of carbamazepine measured in the present study additionally confirmed that biotransformation of carbamazepine occurred in the flumes. Carbamazepine-10,11-epoxide concentrations in the SW reached up to 1.2 mol % of the injected parent mass (Supplementary Fig. S3). In the PW, the highest net-formation was observed in Samplers D at day 42 (Supplementary Fig. S1). But in contrast to the SW concentrations, concentrations on Flowpaths d decreased towards the end of the experiment. That finding contrasts other studies, where the TP showed a constant increase in concentrations in SW and PW and no onset of degradation^23,44^. It is conceivable that the microbial community of the flume sediment developed a function to transform the TP due to long-term exposure of the river sediment. In the PW of River Erpe, carbamazepine-10,11-epoxide concentrations were significantly lower than in the SW, confirming degradation of the TP in the hyporheic zone^39^. Net-formation of the TP hardly occurs in Samplers A, B and C. An interpretation could be that the specific redox or microbial conditions on Flowpath d displayed a niche, where formation of the TP was high and its degradation low. Due to its apparent high persistence in the SW more research on this TP is needed. 10,11-dihydroxy carbamazepine (full name: carbamazepine-10–11-dihydro-10–11-dihydroxy, CAS: 58955-93-4) exhibited an inconsistent pattern in SW and PW concentrations indicating more complex formation-degradation dynamics than its sister compound (Supplementary Fig. S1). The indistinct pattern fits the observation that concentrations of the TP in PW and SW of River Erpe were similar^39^.

### 1H-Benzotriazole and TP 1-methyl-1H-benzotriazole

The corrosion inhibitor 1H-benzotriazole was the third most stable compound in the SW after carbamazepine and clofibric acid, featuring DT50s of 29.6 and 34.8 days in the SW of Flume 1 and Flume 2, respectively^36^. Also in the SW of River Erpe it was found similarly persistent as carbamazepine^53^. Consequently, in the PW of the flumes it resembled the behavior of carbamazepine showing rising DT50s from a to c (Table 2). Only along Flowpath d, 1H-benzotriazole showed highest persistence, whereas carbamazepine was degraded along d similarly to b. Diverse degradation behavior in sediments and aquifers was found previously. In bank filtration studies, 1H-benzotriazole was found persistent under oxic and anoxic conditions^17,56^. In microcosms of aquifer material DT50s of 1H-benzotriazole were lowest under aerobic conditions (43 ± 4.8 d) and up to 83 d under anaerobic conditions^57^. In activated aerobic sludge 1-H-benzotriazole, however, had a DT50 of only 1 day and several biotransformation products were identified^58^. In the sediment of River Erpe 1H-benzotriazole degraded even more rapidly with DT50s of 0.9 to 12.6 h within 40 cm ranging from oxic to suboxic conditions^15^.

In Flumes 1 and 2, the compound’s TP 1-methyl-1H-benzotriazole occurred in the PW of Samplers B, C and D but it was not measured above the LOQ at any point in the SW or in Sampler A. In addition, while concentrations rose similarly along Flowpaths b and c, it was lower along Flowpaths d in both flumes, indicating that net-formation was favored under reducing conditions. After formation, it was degraded within less than 14 days. Interestingly, 1-methyl-1H-benzotriazole was formerly reported as an aerobic TP and rather persistent after formation in oxic activated-sludge batch experiments^58^. And in an oxic aquifer, it was proposed to be formed as a transition product and degraded further to 2-methyl-1H-benzotriazole^59^. However, Liu et al.^57^ found high concentrations of 1-methyl-1H-benzotriazole in aquifer microcosms after 77 days particularly under anaerobic conditions. If the TP is formed predominantly under oxic conditions, our results are in accordance with the study of Liu et al.^57^ indicating that degradation of 1-methyl-1H-benzotriazole also occurs under oxic conditions, rendering it more persistent in more reducing environments. As the compound was not measured above LOQ in the SW, it is apparent that its origin is the hyporheic zone. In the PW of River Erpe, however, 1-methyl-1H-benzotriazole was not detected, which is in agreement with the short formation-degradation cycle observed in the flume sediments^16,39^.

### Acesulfame

Acesulfame DT50s increased from Flowpaths a to b to c, which is in accordance with its sensitivity to oxic conditions reported previously. DT50 on Flowpath d (54.4 h), however, is closer to c (55.0 h) than b (36.6 h), which is contradictory to the assumption that d is similar or even more oxic than b. On Flowpath a (median *k*: 0.11 h^−1^), degradation was in the same order of magnitude as found in a column experiment under oxic and suboxic conditions (0.1 to 0.6 h^−1^)^13^, while Flowpaths b, d and c showed considerably higher DT50s in accordance with the mostly anoxic conditions (Table 2). In the sediment of River Erpe, in-situ DT50s (0.5 to 2.9 h) in depths up to 40 cm were lower than in any of the flowpaths in the present study (6.6 to 55.0 h)^15^. Upon dilution of the sediment taken from River Erpe by 1:10, the community apparently lost some of the degradation capacity. The difference confirms that the bacterial community in the sediment of River Erpe likely adapted well to efficiently degrade acesulfame due to continuous exposure. This kind of adaptation with time has been observed previously^60^. But despite differences in community composition, generally the microbial activity in the original river sediment was likely higher than in the flume sediment, due to higher availability of nutrients and carbon favoring lower DT50s. In both, the river and the flume sediments R was close to 1 indicating negligible retardation of acesulfame^15^. DT50s of acesulfame in the SW were 62.4 h and 48.3 h in Flumes 1 and 2, respectively, which is close to the DT50 on Flowpath c^36^. Acesulfame showed a significant reaction to the amount of bedforms present in the flumes previously^35^. The interpretation that the high redox sensitivity of the compound and the diverse redox environment in the bedforms are responsible for this effect is confirmed by the findings of the present study.

### Bezafibrate and TP 2,4-chlorobenzoic acid

The anti-hyperlipidaemia drug bezafibrate behaved in a similar manner to acesulfame, only with slightly higher DT50s. It was rapidly degraded in the flumes’ SW (DT50s: 2.3 and 2.7 days) and was increasingly degraded in the PW in the succession of Flowpaths c, b and a, indicating a redox sensitivity (Table 2). Similarly to acesulfame, lower degradation on Flowpath d defies the redox pattern. The compounds’ retardation coefficients were comparable as well, ranging between 1.0 and 1.3. The similartity to acesulfame fits also degradation in the Erpe sediment, where DT50s of bezafibrate ranged from 0.8 to 3.7 h, i.e. degradation was about an order of magnitude higher than in the flume sediments^15^.

Similar to 1-methyl-1H-benzotriazole, the TP of bezafibrate, 2,4-chlorobenzoic acid, showed a formation-degradation cycle within the first 14 days after injection. In contrast to the benzotriazole-TP, though, 2,4-chlorobenzoic acid peak concentrations at day 3 were higher in Samplers D than in any other sampler and it was found in the SW. In the SW it appears in relatively high concentrations but not before day 3 and it remains higher than in the PW samplers until day 7, before it dissipates in the SW similar to most of the samplers before day 14 (Supplementary Fig. S2). This dynamic indicates that the TP is generally formed and also dissipated in the PW faster than in the SW. The net-formation was particularly high along Flowpaths d, allowing the interpretation that formation increased behind the oxic zone of Flowpath a, but dissipation outweighed formation in more reduced areas at longer flowpaths towards Samplers B and C. Another interpretation would be that the degrader community in Bedforms 2 are composed in a way that favors formation or hinders dissipation of 2,4-chlorobenzoic acid in contrast to Bedforms 1. Formation-dissipation dynamics of three different TPs of bezafibrate of similar timescale to 2,4-chlorobenzoic acid in the present study were found in aerated sediment–water bottle incubation^44^ and in the PW of a recirculating flume experiment^23^. However, to the best of our knowledge there is no previous study that described formation-dissipation curves of 2,4-chlorobenzoic acid in saturated sediments.

### Valsartan, irbesartan and their TP valsartan acid

Valsartan and irbesartan are both antihypertensive drugs and parts of a group of sartans that have valsartan acid as their main TP^61^. Valsartan DT50s in the SW were 3.7 d in Flume 1 and 3.0 d in Flume 2 and a bit lower than irbesartan with DT50s of 7.5 d in Flume 1 and 5.6 d in Flume 2^36^. In the PW of Flume 1, irbesartan DT50s (3.2 to 93.1 h) were similar to valsartan DT50s (6.4 to 74.1 h) (Table 2). Also the trends between flowpaths were similar for both compounds. They are two of the few compounds, for which distinct differences between flumes were observed. In Flume 1 both compounds showed particularly high degradation on Flowpath b contrasting relatively low degradation along Flowpath d. In Flume 2, however, concentration curves of both compounds were similar in Samplers B and D. The finding may indicate that the sartans were transformed by degraders that were bedform specific in Flume 1 but similarly present in Bedforms 1 and 2 of Flume 2. The finding is intriguing considering the fact that Flumes 1 and 2 have similar hydrological, chemical and bacterial conditions as described above and that very similar behavior in both flumes was found for most other compounds. The reason may be a very specific small scale heterogeneity in chemical or bacterial conditions that sartans are particularly sensitive to. In addition, the finding supports the results of the hydrodynamic model^38^ that Samplers B and C are not positioned on overlapping flowpaths, otherwise the difference in concentrations in B and C would be difficult to interpret (Figs. S7, S8).

More interestingly, the common TP valsartan acid, in contrast to its parent compounds, exhibited a clear similarity in behavior between Flumes 1 and 2 and a higher net-formation on Flowpaths d compared to b (Fig. 2). Therefore, conditions favoring valsartan acid formation were (1) bedform-specific and not flume-specific and (2) cannot be inferred from the degradation behavior of its parent compounds. The discrepancy between irbesartan and valsartan degradation and valsartan acid formation, respectively, allows for two interpretations. Firstly, the single transformation steps may be mediated by differing bacterial species. Transformation of both parent compounds likely starts by oxidative dealkylation to valsartan acid precursors. The bacterial community responsible for this step, thus, may have caused the particular pattern observed for irbesartan and valsartan. The final step to valsartan acid formation is an aldehyde oxidation in the case of irbesartan and an oxidation/hydrolysis step for valsartan^36,62^. The bacterial community responsible for these steps may have been bedform-, but not flume-specific. Another observation is noteworthy: The concentrations of valsartan acid in the PW hardly ever exceeded the concentrations in the SW (except for Sampler D in Flume 1 on days 1 and 28 and Sampler D in Flume 2 on day 78). Although valsartan acid has been generally reported as quite persistent to biodegradation, it is therefore conceivable that it degraded in the PW of Bedforms 1 reaching a formation-degradation equilibrium responsible for the steady concentrations. In this case, the reason for the discrepancy between concentration patterns of parents and TP could be that communities involved in valsartan acid degradation were bedform- but not flume-specific and outweighed the effect of the sampler-specific parent degraders. Valsartan acid was previously found to form in the SW and PW of River Erpe and was reported to be relatively persistent under various redox conditions^15,16,53,61^. However, in a different study high valsartan acid degradation was observed in bank filtration under oxic conditions^13^.

### Metoprolol, sotalol and metoprolol acid

In both flumes, the β-blocker metoprolol was completely removed in SW and PW before the first sampling after injection at day 1. Atenolol, the second parent compound of metoprolol acid, showed a corresponding behavior in the SW and in Sampler B. Thus, atenolol concentrations in the other samplers were presumably below LOQ as well. The high degradation rates of the β-blockers are attributed to the high bacterial diversity in the group of treatments both Flumes 1 and 2 were part of^36^. Sotalol, a β-blocker of similar structure to metoprolol and atenolol also degraded rapidly, but concentrations above LOQ were measurable in SW and PW up until day 7 in Sampler C (Supplementary Fig. S2). The DT50s in the SW were 0.8 and 0.7 days in Flumes 1 and 2, respectively. While sotalol concentrations were still above 4 µg L^−1^ at day 1 in the SW, they never reached more than 1.3 µg L^−1^ in the PW, indicating rapid degradation in the sediment. DT50s were lowest on Flowpath a (0.67 h) and highest on Flowpath c (12.0 h), resembling the decreasing degradation with longer flowpaths in sediment of river Erpe. In contrast to other compounds, degradation of sotalol was in the same order of magnitude as estimated in the sediment of River Erpe with DT50s of 0.8 to 5.8 h^15^.

Metoprolol acid, a main TP of metoprolol and atenolol (not sotalol) showed measurable formation-degradation dynamics in the first 7 days in SW and PW. In agreement with the rapid disappearance of its parent compounds, the TP was readily present in the SW of Flume 1 at day 1. This pattern contrasts the other TPs which first formed in the PW (e.g. 1-methyl-1H-benzotriazole) or appeared later (e.g. valsartan acid). Metoprolol acid thereafter behaved like a parent compound in Flume 1, migrating from Sampler A over B/D to C and degraded. Several second-generation TPs were detected in the SW of the flumes, confirming that metoprolol acid is a transient product in the degradation pathway of metoprolol^36^. In addition, metoprolol acid is the only compound of the present study for which a clear difference between Flume 1 and Flume 2 occurred. In Flume 1, the concentrations in the SW reached 1.4 µg L^−1^ and more than 0.7 µg L^−1^ in Samplers A, B, D and C. Concentrations in SW and PW of Flume 2 remained below 0.3 µg L^−1^. Metoprolol acid was previously shown to be formed from atenolol by hydrolysis mediated by the common freshwater cyanobacteria *Synechococcus sp.* and from metoprolol by oxidation by *Chlamydomonas reinhardtii*, a green algal species^63^. Moreover, Cytochrome p450 mediated dealkylation of metoprolol is common in human metabolism^64^ and cyanobacteria have an extensive catalogue of the Cytochrome p450 monooxygenases^65^. Hence, the higher presence of cyanobacteria in Flume 2 (Fig. 4) might have played a major role not only in formation, but also in the quick metoprolol acid degradation. Another indication for the role of cyanobacteria is that in the sediment of River Erpe, where relative abundance of cyanobacteria was lower than in the flumes^49^, metoprolol was present in measurable amounts down to 40 cm^15^.

Metoprolol acid and valsartan acid both showed high concentrations and high formation in the SW and PW of River Erpe^15,53^ but both TPs clearly differ in their behavior in the flume sediments. Besides its lower persistence, metoprolol acid was strongly sensitive to differences between the flumes and behaved similarly in Bedforms 1 and 2, while valsartan acid was only sensitive to differences between bedforms. Nodler et al.^66^ also observed high differences in formation patterns of both TPs, attributing it to their high sensitivity to small changes in microbial communities^49^.

### Venlafaxine and O-desmethylvenlafaxine

DT50s of venlafaxine increased in the order of a, b, d and c and, thus, indicate redox sensitivity of the compound. In the sediment of River Erpe, in contrast, venlafaxine was not significantly removed^15^. The DT50 on Flowpath a (0.97 h) was indeed one of the lowest values estimated, however, the fit of the curve was relatively poor, likely attributable to a particularly low concentration on day 14, which might be considered an outlier. Therefore, degradation on Flowpath a might have been overestimated. The high *R* obtained for that flowpath might be plausible due to the relatively late breakthrough of venlafaxine (Supplementary Fig. S8). DT50s in the SW were an order of magnitude higher than in the PW, with 5.2 d in Flume 2 and 5.0 d in Flume 1^36^.

O-Desmethylvenlafaxine displayed concentrations of up to 0.3 µg L^−1^ in the PW already at day 0 meaning that the TP was present in the PW prior to injection of micropollutants and, thus, derived from the Erpe sediment. Although this finding confirms a high stability of the compound in the Erpe sediment as discussed in Schaper et al.^15^, within the duration of the flume experiment O-desmethylvenlafaxine degraded almost completely (Supplementary Fig. S2).

### Metformin and sitagliptin

Metformin, an anti-diabetic drug, showed lowest DT50 on Flowpath c (20 h) in contrast to most other compounds. On Flowpaths d and b, DT50s were highest (Fig. 6). Hence, the compound does not have a trend following redox conditions. An explanation could be that the long retention time in addition to high retardation favored the high degradation on Flowpath c. Concentrations between flumes and bedforms match very well for metformin (Fig. 2). In the sediment of River Erpe, DT50s were lower (1.1–3.9 h). Similar to the Erpe sediment, metformin was one of the compounds featuring the highest retardation in the flumes^15^. In a large-scale flume experiment investigating the fate of metformin in the hyporheic zone of dunes, metformin displayed DT50s in the same order of magnitude as in the present study^49^. However, the compound was degraded mostly in the stoss side of the dunes. The finding contradicts the results of the present study. It appears that redox conditions or the retention time of flowpaths are a poor predictor for metformin turnover. The reason for the differences might instead be found in the microbial composition along flowpaths, as high susceptibility of metformin to variations in the bacterial community composition has been observed before^67^. However, on all flowpaths, DT50s were considerably lower than in the SW (4.3 and 4.4 days) confirming that degradation of metformin primarily takes place in the hyporheic zone as previously suggested. Sitagliptin, also an anti-diabetic drug, which is often taken in combination with metformin, showed degradation similar to sotalol following the trend expected for redox-sensitive compounds. However, concentrations in the PW were even lower than for sotalol hardly displaying breakthrough curves. Consequently, posteriors of *R* were relatively wide.

The major TP of metformin, guanylurea, was not detected in the SW or PW of Flumes 1 and 2. In the SW of other flumes of lower bacterial diversity in the same experiment, the TP was found^36^, which indicates that the bacterial community in the flumes of the present study does not resist guanylurea formation but rather promotes rapid degradation inhibiting detection within the sampling interval of the experiment. This occurs under all conditions of all flowpaths of the study.

### Flowpath specific degradation behaviour

The majority of DT50s estimated in the flume sediment are lower than for the same compounds in the SW^36^. It indicates that most compounds were primarily degraded in the hyporheic zone and highlights its importance for stream water quality. DT50s measured in the sediment of River Erpe in-situ are mostly slightly lower than in the flume sediments, which was expected considering the 1:10 dilution of the original sediment in the flumes and the low organic carbon content.

Many of the compounds investigated in the present study showed degradation trends following decreasing redox potential and increasing travel times along Flowpaths a, b and c, namely acesulfame, bezafibrate, ibuprofen, naproxen, irbesartan, valsartan, sitagliptin, sotalol and venlafaxine. However, of this group only venlafaxine, sotalol and sitagliptin, which were at the same time the most reactive compounds, degraded on Flowpath d similarly to Flowpath b as expected for redox-sensitive compounds. The rest of the group degraded considerably less on Flowpath d than expected. The reactions of other compounds were more complex. Metformin and sulfamethoxazole, for instance, showed highest degradation on the most reductive Flowpath c, diclofenac degraded best on Flowpath b. Particularly intriguing was the behavior of the TPs. While no parent compounds showed highest degradation on Flowpath d, many TPs such as valsartan acid, carbamazepine-10,11-epoxide and 2/4-chlorobenzoic acid showed higher net-formation on d than on any other flowpath. Hence, Bedforms 1 and 2, despite their strong similarity in most boundary conditions, had a considerably different potential for compound transformation. This phenomenon cannot just be explained as a strong outlier of a single flowpath, as concentration curves in Samplers D of both flumes were mostly very similar and closer to each other than to concentrations in Sampler B of the same flume. The differences between bedforms were often higher than the differences between the two flumes. One likely explanation of the general peculiarity of behavior on Flowpath d is a potential general deviation of the microbial community composition between Bedforms 1 and 2 induced by slightly differing hydrodynamic characteristics and flow fields due to their different positions in the flumes.

The boundary conditions in both flumes were generally very similar and most concentration curves of the same samplers in different flumes displayed substantially high similarity between flumes. Only three compounds deviated from this observation. Metoprolol acid showed concurrent curves between bedforms, but especially low net-formation in Flume 2, potentially attributable to higher presence of cyanobacteria in that flume sediment. Irbesartan and valsartan, while displaying behavior very similar to each other, reacted both flume- and bedform specific. In Flume 1, degradation of the parent compounds on Flowpath b was particularly high, contrasting the formation dynamics of the common TP valsartan acid which behaved similarly in both Flumes.

### Relevance of controlling factors

Hence in general, as expected for short, oxic flowpaths, Flowpath a showed the highest parent compound degradation potential. This confirms, that redox zonation is a strong controlling factor for many compounds. However, some compounds of high abundance and relevance, such as valsartan, irbesartan and metformin, showed very individual behavior. More importantly, net-formation of potentially persistent TPs appeared to be independent of the redox milieus. The specific behavior of TPs along Flowpaths d cannot be fully explained within the scope of the study, but differences in the microbial community between bedforms are likely the reason. It has been shown recently that many compounds are sensitive to overall microbial diversity in flume and river sediments^36,41,67^. Therefore, it is plausible that some are also sensitive to small-scale differences between bedforms or even along single flowpaths. An interpretation why TPs and parent compounds react very differently is that parent compounds can be transformed by a cascade of reactions and thus potentially by a more diverse set of species, while for formation and potential degradation of TPs, more specialised species are responsible potentially occurring in high small-scale heterogeneity in the sediments.

The behavior of the beta-blockers and metoprolol acid, as well as the behavior of the sartans in the flume sediments show that compounds of structural similarity are likely susceptible to similar microbial community compositions. The influence of microbial diversity and community composition on the fate of micropollutants is still understudied. The results of the present study show that for the fate of many relevant compounds and particularly for formation of transformation products, heterogeneity of sediment microbial diversity on a centimeter-scale is likely a major controlling factor even outweighing the influence of redox zonation. High similarities between the flumes indicate furthermore, that the hydrodynamic flow field in the sediment has a strong influence on the microbial community. Therefore, both factors, the flow field and the community composition in combination, are potentially the most relevant drivers for formation of transformation products in the hyporheic zone. The relation of hyporheic flow fields and microbial community composition and activity, as well as the subsequent impact on transformation of micropollutants needs further research. Our study demonstrates that flume experiments are useful tools to systematically examine these interactions.

## Supplementary Information

Supplementary Information.
