# Author Correction: Transformation of organic micropollutants along hyporheic flow in bedforms of river-simulating flumes

**DOI:** 10.1038/s41598-021-04180-0

**Published:** 2021-12-20

**Authors:** Anna Jaeger, Malte Posselt, Jonas L. Schaper, Andrea Betterle, Cyrus Rutere, Claudia Coll, Jonas Mechelke, Muhammad Raza, Karin Meinikmann, Andrea Portmann, Phillip J. Blaen, Marcus A. Horn, Stefan Krause, Jörg Lewandowski

**Affiliations:** 1 Department Ecohydrology, Leibniz Institute of Freshwater Ecology and Inland Fisheries, Berlin, Germany; 2 Geography Department, Humboldt University Berlin, Berlin, Germany; 3 Department of Environmental Science, Stockholm University, Stockholm, Sweden; 4 Center for Applied Geoscience, Eberhard Karls University of Tübingen, Tübingen, Germany; 5 Department of Civil, Environmental and Mechanical Engineering, University of Trento, Trento, Italy; 6 Department of Ecological Microbiology, University of Bayreuth, Bayreuth, Germany; 7 Eawag, Swiss Federal Institute of Aquatic Science and Technology, Dübendorf, Switzerland; 8 Institute of Biogeochemistry and Pollutant Dynamics, ETH Zürich, Zürich, Switzerland; 9 Institute of Applied Geosciences, Technical University of Darmstadt, Darmstadt, Germany; 10 IWW Water Centre, Mülheim an der Ruhr, Germany; 11 Julius Kühn Institute – Federal Research Centre for Cultivated Plants, Institute for Ecological Chemistry, Plant Analysis and Stored Product Protection, Berlin, Germany; 12 Civil and Environmental Engineering, Colorado School of Mines, Golden, CO USA; 13 School of Geography, Earth and Environmental Sciences, University of Birmingham, Birmingham, UK; 14 Yorkshire Water, Leeds, UK; 15 Institute of Microbiology, Leibniz University of Hannover, Hannover, Germany; 16 Université Claude Bernard Lyon 1, Ecologie des Hydrosystèmes Naturels et Anthropisés (LEHNA), Villeurbanne, France

Correction to: *Scientific Reports* https://doi.org/10.1038/s41598-021-91519-2, published online 22 June 2021

The original version of this Article contained an error in Affiliation 11, which was incorrectly given as ‘Julius Kühn Institute – Federal Research Centre for Cultivated Plants, Institute for Breeding Research on Agricultural Crops, Berlin, Germany’. The correct affiliation is listed below.

Julius Kühn Institute – Federal Research Centre for Cultivated Plants, Institute for Ecological Chemistry, Plant Analysis and Stored Product Protection, Berlin, Germany.

The original Article has been corrected.

