# Supplementary material for: Transformation of organic micropollutants along hyporheic flow in bedforms of river-simulating flumes
Source: Sci Rep. 2021 Jun 22;11:13034. doi: 10.1038/s41598-021-91519-2 (PMC8219703; doi:10.1038/s41598-021-91519-2)
Supplement: Supplementary file 1 — Supplementary Information. [file 41598_2021_91519_MOESM1_ESM.pdf]

Supplementary Information

Transformation of organic micropollutants along hyporheic flow in bedforms of river-simulating flumes

Anna Jaeger (a,b)*, Malte Posselt (c), Jonas L. Schaper (d), Andrea Betterle (e), Cyrus Rutere (f), Claudia Coll (c), Jonas Mechelke (g,h), Muhammad Raza (i,k), Karin Meinikmann (l), Andrea Portmann (m), Phillip J. Blaen (n,o), Marcus A. Horn (f,p), Stefan Krause (n,q), and Jörg Lewandowski (a,b)

a) Department Ecohydrology, Leibniz Institute of Freshwater Ecology and Inland Fisheries, Berlin, Germany

b) Geography Department, Humboldt University Berlin, Berlin, Germany

c) Department of Environmental Science, Stockholm University, Stockholm, Sweden

d) Center for Applied Geoscience, Eberhard Karls University of Tübingen, Tübingen, Germany

e) Department of Civil, Environmental and Mechanical Engineering, University of Trento, Trento, Italy

f) Department of Ecological Microbiology, University of Bayreuth, Bayreuth, Germany

g) Eawag, Swiss Federal Institute of Aquatic Science and Technology, Dübendorf, Switzerland

h) Institute of Biogeochemistry and Pollutant Dynamics, ETH Zürich, Zürich, Switzerland

i) Institute of Applied Geosciences, Technical University of Darmstadt, Darmstadt, Germany

k) IWW Water Centre, Mülheim an der Ruhr, Germany

l) Julius Kühn Institute – Federal Research Centre for Cultivated Plants, Institute for Breeding Research on Agricultural Crops, Berlin, Germany

m) Civil and Environmental Engineering, Colorado School of Mines, Golden, Colorado, USA

n) School of Geography, Earth and Environmental Sciences, University of Birmingham, Birmingham, UK

o) Yorkshire Water, Leeds, UK

p) Institute of Microbiology, Leibniz University of Hannover, Hannover, Germany

q) Université Claude Bernard Lyon 1, Ecologie des Hydrosystèmes Naturels et Anthropisés (LEHNA), Villeurbanne, France

*corresponding author, email: anna.jaeger@igb-berlin.de

Table S1 Nutrient mixes added to the flume SW

substance	mass added per flume		target concentration		Day added	vendor
$CaCl_2$	15285	mg	254.7	mg L^{-1}	-12	Fisher scientific UK
$NaHCO_3$	1498	mg	25	mg L^{-1}	-12	Sigma-Aldrich
KCl	2722	mg	45.4	mg L^{-1}	-12	BDH VWR Chemicals
$MgSO_4$*7H_2O	6583	mg	109.7	mg L^{-1}	-12	BDH VWR Chemicals
Na_2SO_4	6852	mg	114.2	mg L^{-1}	-12	Fisher scientific UK
KH_2PO_4	125	mg	2.1	mg L^{-1}	-12	Fisher scientific UK
NH_4Cl	1719	mg	28.6	mg L^{-1}	-12	BDH VWR Chemicals
$C_6H_{12}O_6$	9000	mg	150	mg L^{-1}	-12	Fisher scientific UK
$MnCl_2$*4$H_2$0	472	mg	7.9	mg L^{-1}	-12	Sigma-Aldrich
$NaNO_3$	9289	mg	154.8	mg L^{-1}	-12	Fisher scientific UK
$CuSO_4$	58	µg	0.96	µg L^{-1}	-12	VWR International
Na_2SeO_3	23	µg	0.38	µg L^{-1}	-12	VWR International
Na_2Mo_4 *H_2O	62	µg	1.03	µg L^{-1}	-12	VWR International
Na_2WO_4*2H_2O	178	µg	2.97	µg L^{-1}	-12	VWR International
$NiCl_2$	163	µg	2.72	µg L^{-1}	-12	VWR International
H_3BO_3	360	µg	6	µg L^{-1}	-12	VWR International
$ZnCl_2$	430	µg	7.16	µg L^{-1}	-12	VWR International
$CoCl_2$	324	µg	5.4	µg L^{-1}	-12	VWR International
ribovlavin	30	µg	0.5	µg L^{-1}	-12	VWR International
biotin	300	µg	5	µg L^{-1}	-12	VWR International
folic acid	300	µg	5	µg L^{-1}	-12	VWR International
nicotinic acid	300	µg	5	µg L^{-1}	-12	VWR International
pantothenic acid	300	µg	5	µg L^{-1}	-12	VWR International
pyridoxal-HCl	300	µg	5	µg L^{-1}	-12	VWR International
thiamine	300	µg	5	µg L^{-1}	-12	VWR International
choline chloride	300	µg	5	µg L^{-1}	-12	VWR International
myoinositol	600	µg	10	µg L^{-1}	-12	VWR International
vitmain B12	1	µg	0.01	µg L^{-1}	-12	VWR International
lipoic acid	376	µg	6.26	µg L^{-1}	-12	VWR International
p-aminobenzoic acid	376	µg	6.26	µg L^{-1}	-12	VWR International
NH_4Cl	2315.6	mg	38.6	mg L^{-1}	10	BDH VWR Chemicals
NH_4NO_3	690.88	mg	11.5	mg L^{-1}	46	n.a.
KH_2PO_4	66.72	mg	1.1	mg L^{-1}	46	Fisher scientific UK

Table S2 Compounds measured at Stockholm University according to Posselt, et al. [1] and Posselt, et al. [2] and associated compound properties

Compound	parent or TP	parent name	LOQ [µg L^{-1}]	% of samples above LOQ	log Dow	logKow	mol. Mass [g mol^{-1}]	Speciation	CAS
10,11-Dihydroxy carbamazepine*	TP	Carbamazepine	0.086	36.4	0.81	0.81	270.3	N	58955-93-4
1H-Benzotriazole	parent		0.18	100	1.19	1.26	119.1	N	95-14-7
1-Hydroxyibuprofen	TP	Ibuprofen	1.358	0.0	-0.64	2.69	222.3	A	53949-53-4
1-Methyl-1H-Benzotriazole	TP	1H-Benzotriazole	0.126	30.7	1.42	1.42	133.2	N	13351-73-0
2/3-Hydroxyibuprofen	TP	Ibuprofen	2.836	0.0	-0.92	2.37	222.3	A	51146-55-5/53949-54-5
2/4-Chlorobenzoic acid	TP	Bezafibrate	0.306	21.6	-1.29	2.23	156.6	A	118-91-2/74-11-3
4-Hydroxy-1H-Benzotriazole	TP	1H-Benzotriazole	0.463	0.0	0.3	1	135.1	A	na
4-Hydroxydiclofenac	TP	Diclofenac	0.006	38.6	0.3	3.96	312.1	A	64118-84-9
Acesulfame	parent		0.049	36.4	-1.49	-0.55	163.2	A	55589-62-3
Acridine	TP	Carbamazepine	143	0.0	3.5	3.51	179.2	N	260-94-6
Acridone	TP	Carbamazepine	419	0.0	4.2	4.2	195.2	N	578-95-0
alpha-Hydroxymetoprolol	TP	Metoprolol	0.632	0.0	-0.54	0.84	283.4	C	56392-16-6
Bezafibrate	parent		0.099	37.5	0.5	3.99	361.8	A	41859-67-0
Carbamazepine	parent		0.055	100	2.77	2.77	236.3	N	298-46-4
Carbamazepine-10,11-epoxide	TP	Carbamazepine	0.036	26.1	1.97	1.97	252.3	N	36507-30-9
Carboxyibuprofen	TP	Ibuprofen	1.661	0.0	-3.95	2.78	236.3	A	15935-54-3
Chlorothiazide	TP	Hydrochlorothiazide	0.084	72.7	-0.49	-0.44	295.7	N	58-94-6
Clofibric acid	parent		0.069	90.9	-0.61	2.9	214.6	A	882-09-7
Diclofenac	parent		0.535	77.3	0.8	4.26	296.1	A	15307-86-5
Diclofenac amide	TP	Diclofenac	0.166	0.0	3.8	3.8	278.1	N	15362-40-0
Furosemide	parent		0.565	59.1	-1.68	1.75	330.7	A	54-31-9
Gemfibrozil	parent		0.075	86.4	1.02	4.39	250.3	A	25812-30-0
Guanylurea	TP	Metformin	0.541	0.0	-3.37	-2.03	102.1	C	141-83-3
Hydrochlorothiazide	parent		0.285	97.7	-0.64	-0.58	297.7	N	58-93-5
Ibuprofen	parent		1.571	26.1	0.66	3.84	206.3	A	15687-27-1
Irbesartan	parent		0.084	77.3	3.99	5.39	428.5	A	138402-11-6
Ketoprofen	parent		0.223	53.4	0.14	3.61	254.3	A	22071-15-4
Metformin	parent		0.473	39.8	-3.66	-1.36	129.2	C	657-24-9
Metoprolol	parent		0.178	1.1	0.38	1.76	267.4	C	37350-58-6
Metoprolol acid	TP	Metoprolol	0.086	29.5	-1.26	-1.24	267.3	Z	56392-14-4
Naproxen	parent		0.385	44.3	-0.44	2.99	230.3	A	22204-53-1
O-Desmethylvenlafaxine	TP	Venlafaxine	0.034	78.4	1.88	2.29	263.4	C	93413-62-8
Propranolol	parent		0.059	2.3	1.2	2.58	259.3	C	525-66-6
Sitagliptin	parent		0.026	96.6	0.65	1.26	407.3	C	486460-32-6
Sotalol	parent		0.051	26.1	-1.28	-0.4	272.4	C	3930-20-9
Sulfamethoxazole	parent		0.054	83.0	-0.13	0.79	253.3	A	723-46-6
Sulfamethoxazole β-D-glucuronide	TP	Sulfamethoxazole	0.459	1.1	-4.59	-1.3	429.4	A	14365-52-7
Valsartan	parent		0.144	37.5	0.34	5.27	435.5	A	137862-53-4
Valsartan acid	TP	Valsartan and Irbesartan	0.774	76.1	-1.83	3.18	266.3	A	164265-78-5
Venlafaxine	parent		0.025	94.3	2.04	2.74	277.4	C	93413-69-5

* full name: Carbamazepine-10-11-dihydro-10-11-dihydrox

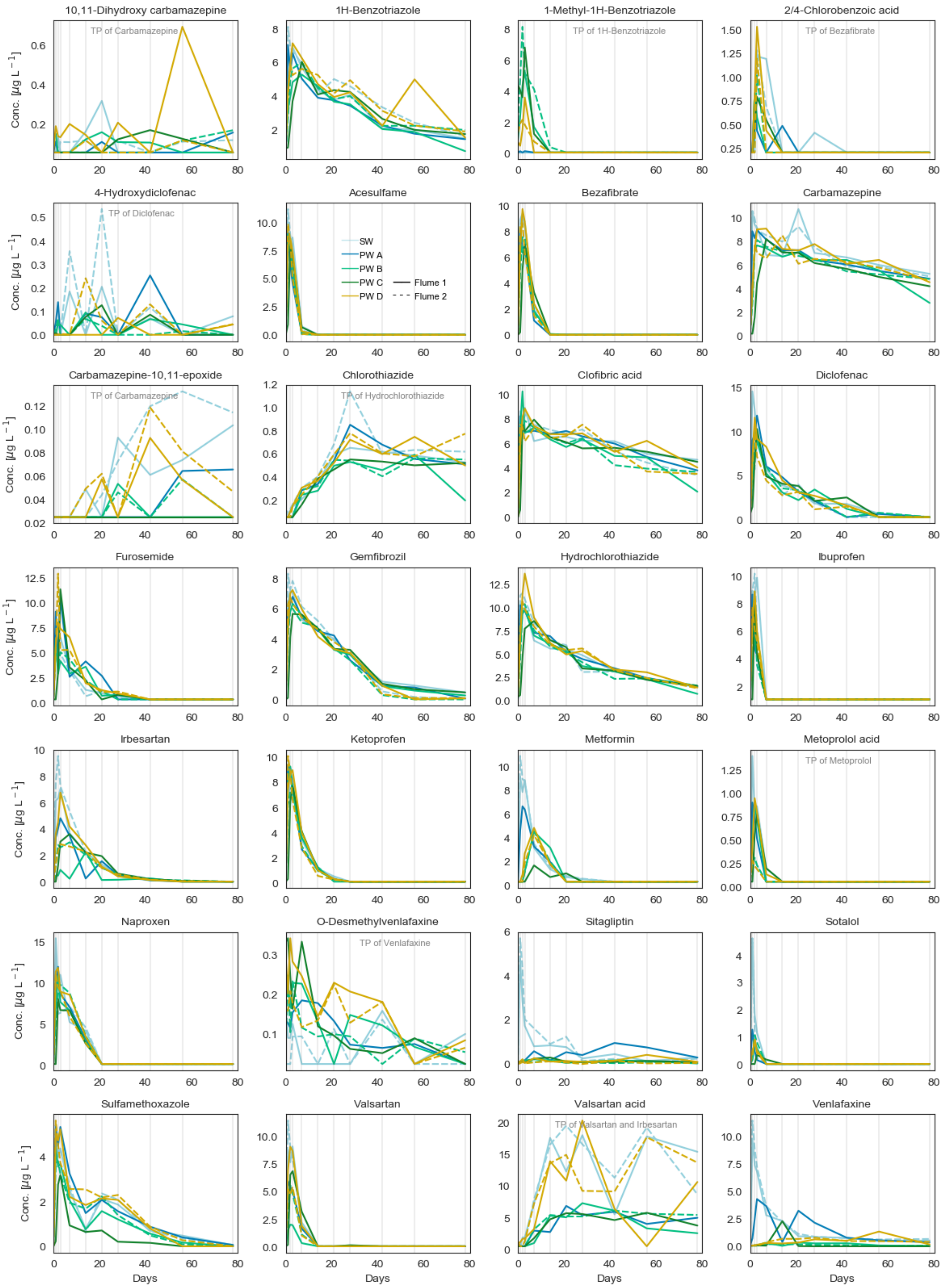

Figure S1 Measured concentrations day 0 to day 78 in the SW, in PW Samplers A, B, C and D in Flume 1 and PW Samplers B and D in Flume 2 of all compounds displaying >5% of all concentrations above LOQ. Grey vertical lines indicate sampling days.

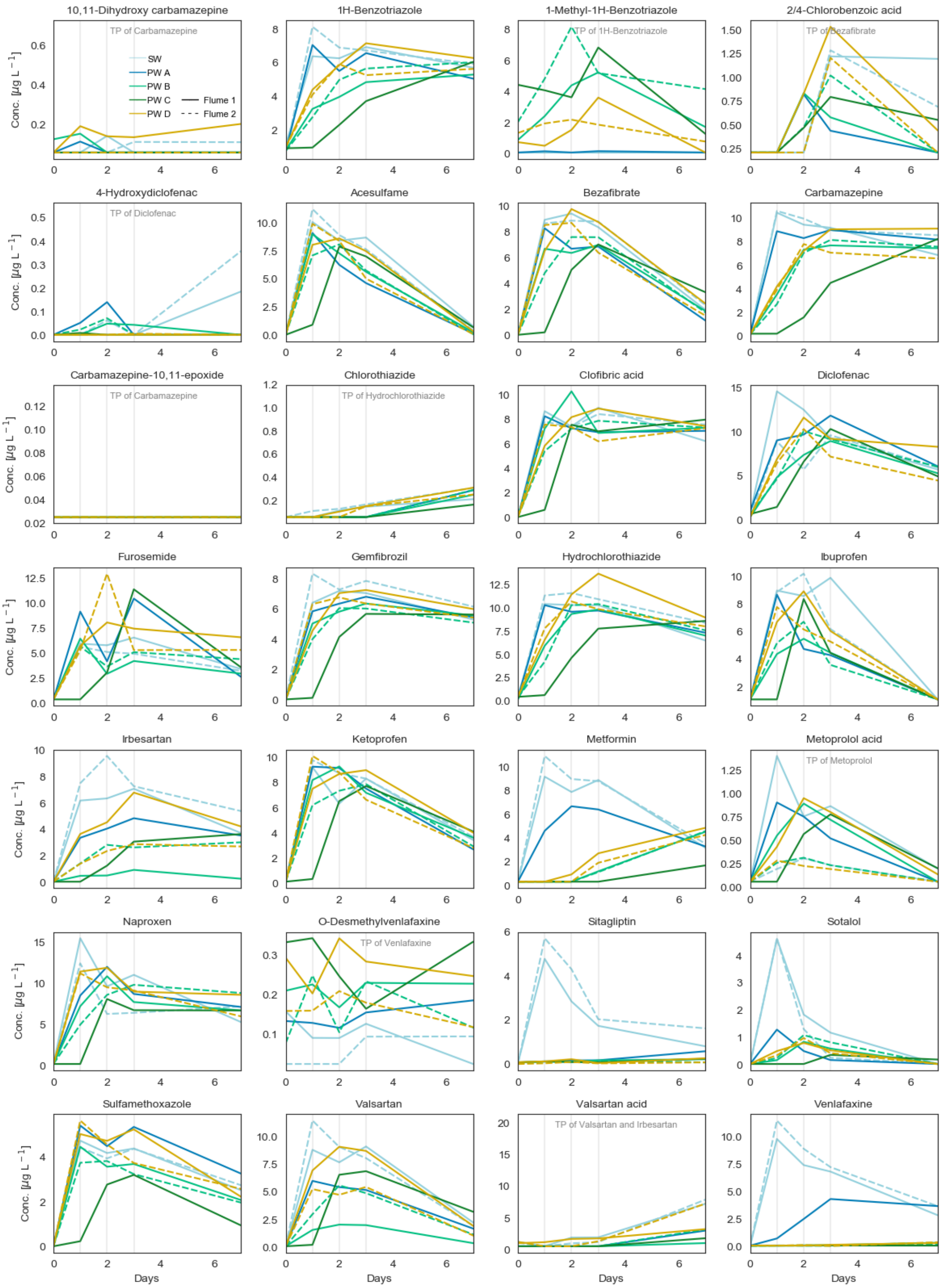

Figure S2 Measured concentrations day 0 to day 7 (break through curves) in the SW, in PW Samplers A, B, C and D in Flume 1 and PW Samplers B and D in Flume 2 of all compounds displaying >5% of all concentrations above LOQ. Grey vertical lines indicate sampling days.

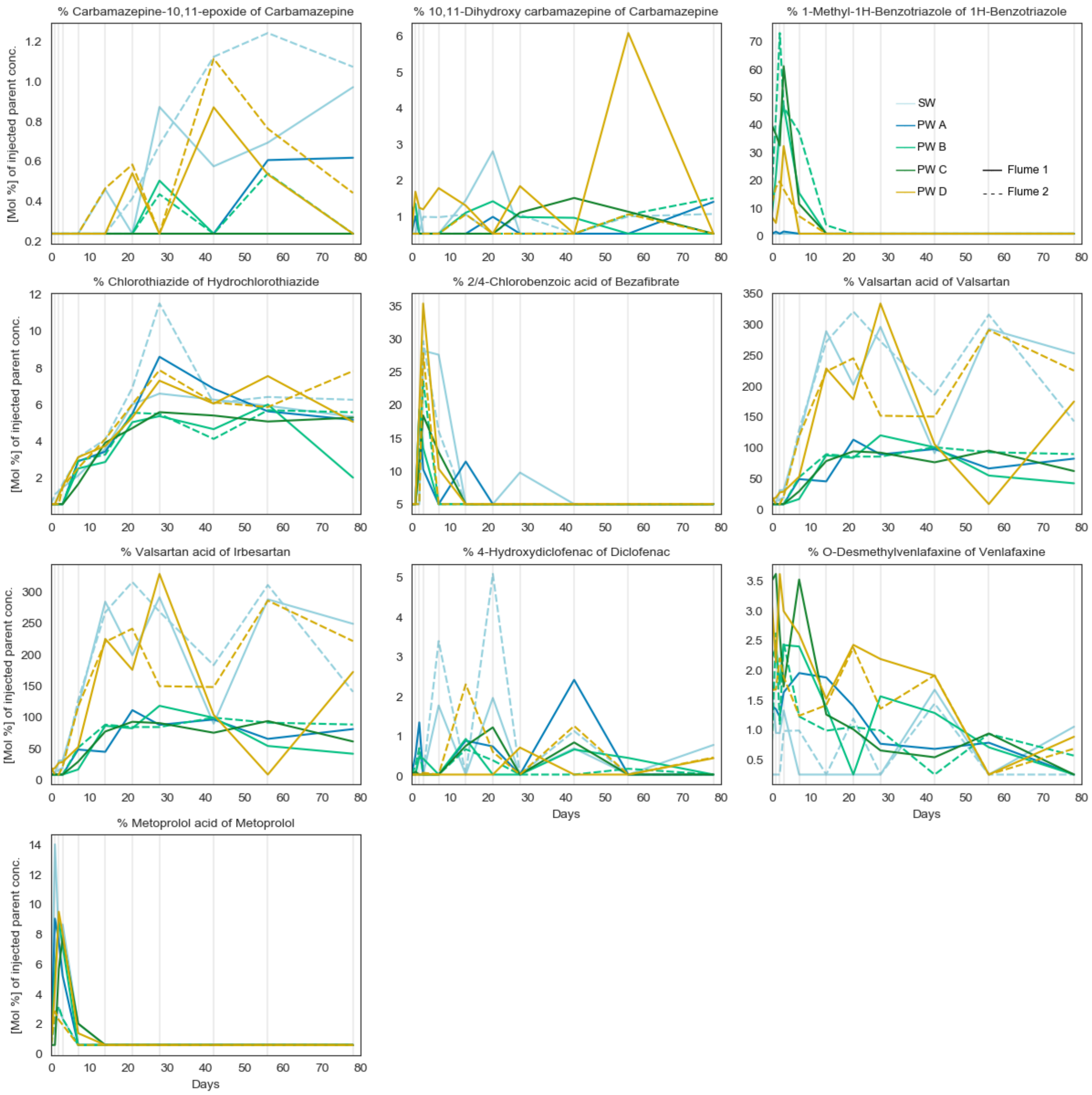

Figure S3 Share of TP of total injected mols of parent compounds in the SW, in PW Samplers A, B, C and D in Flume 1 and PW Samplers B and D in Flume 2. Grey vertical lines indicate sampling days.

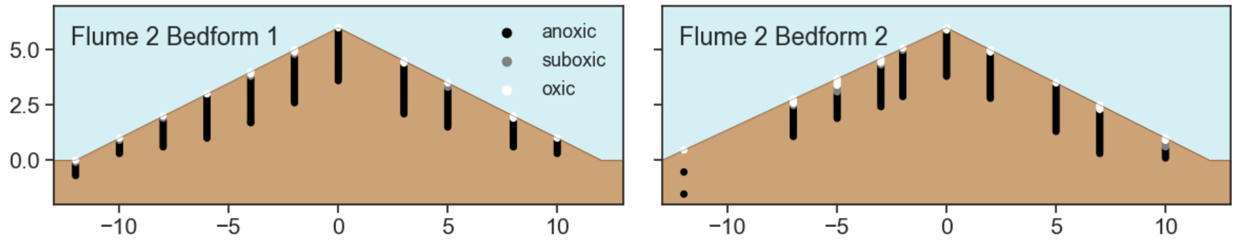

Figure S4 Longitudinal cross sections of Bedforms 1 and 2 in Flume 2 (spatial coordinates in cm). Each dot represents one oxygen concentrations measurement, the color indicates the oxygen zones (white: oxic: >1 mg L^{-1} O_2; grey: suboxic 0.01 - 1 mg L^{-1} O_2; black: anoxic: <0.01 mg L^{-1} O_2). At the time of the measurement the upstream side of Bedform 2 showed a slightly elongated slope of 13 cm, which is why the bedform is not exactly symmetrical in this illustration.

Table S3 Medians of reactive model posterior distributions for degradation rate constants (*k*), half-lives (DT50s) and retardation coefficients (*R*) and respective inter quartile ranges (in brackets) on Flowpaths a, b, c and d of Flume 1. DT50s exceeding the DT50 thresholds (see Table S4) were set to infinity (inf).

**Compound**	**Retardation coefficient R [-]**				**Half-life DT50 [h]**				**Degradation rate constant k [h^{-1}]**			
	**a**	**b**	**c**	**d**	**a**	**b**	**c**	**d**	**a**	**b**	**c**	**d**
1H-Benzotriazole	1.76 (1.14)	1.91 (0.22)	1.90 (0.11)	1.41 (0.09)	53.5 (39.5)	97.3 (42.8)	287 (208)	Inf (335 (450))	0.013 (0.009)	0.007 (0.003)	0.002 (0.002)	0.002 (0.002)
Acesulfame	1.10 (0.13)	1.00 (0.00)	1.00 (0.00)	1.00 (0.01)	6.63 (0.58)	36.62 (2.45)	54.97 (3.34)	54.35 (5.65)	0.105 (0.009)	0.019 (0.001)	0.013 (0.001)	0.013 (0.001)
Bezafibrate	1.09 (0.14)	1.01 (0.02)	1.28 (0.03)	1.05 (0.05)	7.43 (1.05)	36.94 (4.29)	87.49 (13.65)	92.4 (25.9)	0.093 (0.013)	0.019 (0.002)	0.008 (0.001)	0.007 (0.002)
Carbamazepine	2.63 (1.31)	1.61 (0.11)	1.98 (0.06)	1.39 (0.07)	49.0 (39.4)	106 (39.2)	285 (196)	85.09 (26.63)	0.014 (0.010)	0.007 (0.002)	0.002 (0.002)	0.008 (0.003)
Clofibric acid	2.53 (0.84)	1.02 (0.02)	1.17 (0.02)	1.23 (0.04)	Inf (391 (691))	Inf (739 (868))	Inf (1920 (3001))	Inf (1943 (3232))	0.002 (0.003)	0.001 (0.001)	<0.0005	<0.0005
Diclofenac	5.05 (1.11)	1.39 (0.09)	1.11 (0.03)	1.17 (0.05)	Inf (186 (248))	44.34 (5.15)	139 (29.9)	143 (53.0)	0.004 (0.004)	0.016 (0.002)	0.005 (0.001)	0.005 (0.002)
Furosemide	7.58 (9.09)	1.17 (0.28)	1.42 (0.16)	1.26 (0.16)	Inf (61.5 (112))	36.96 (10.77)	163 (125)	Inf (718 (1172))	0.011 (0.017)	0.019 (0.005)	0.004 (0.003)	0.001 (0.001)
Gemfibrozil	2.62 (0.61)	1.12 (0.07)	1.35 (0.03)	1.31 (0.03)	Inf (279 (400))	154 (39.8)	273 (76.6)	196 (71.3)	0.002 (0.003)	0.005 (0.001)	0.003 (0.001)	0.004 (0.001)
Hydrochlorothiazide	1.29 (0.44)	1.13 (0.08)	1.41 (0.04)	1.21 (0.03)	Inf (846 (1518))	Inf (712 (756))	Inf (4375 (6828))	Inf (5773 (9909))	0.001 (0.001)	0.001 (0.001)	<0.0005	<0.0005
Ibuprofen	5.62 (5.04)	1.20 (0.24)	1.07 (0.11)	1.11 (0.13)	3.71 (1.31)	24.74 (6.12)	53.55 (15.75)	58.29 (31.00)	0.187 (0.066)	0.028 (0.007)	0.013 (0.004)	0.012 (0.006)
Irbesartan	1.53 (0.83)	2.42 (0.23)	1.91 (0.04)	1.38 (0.03)	3.25 (0.32)	5.06 (0.20)	93.10 (10.67)	75.56 (11.85)	0.213 (0.021)	0.137 (0.005)	0.007 (0.001)	0.009 (0.001)
Ketoprofen	1.30 (0.42)	1.03 (0.04)	1.30 (0.05)	1.04 (0.06)	21.06 (7.91)	Inf (210 (168))	63.52 (10.55)	75.74 (26.53)	0.033 (0.012)	0.003 (0.002)	0.011 (0.002)	0.009 (0.003)
Metformin	10.6 (1.07)	8.03 (0.28)	4.31 (0.17)	4.46 (0.09)	28.33 (6.56)	136 (50.2)	20.03 (0.71)	36.65 (3.39)	0.024 (0.006)	0.005 (0.002)	0.035 (0.001)	0.019 (0.002)
Naproxen	4.76 (1.05)	1.05 (0.06)	1.03 (0.04)	1.01 (0.02)	30.14 (12.86)	65.4 (12.9)	67.68 (8.24)	Inf (200 (135))	0.023 (0.010)	0.011 (0.002)	0.010 (0.001)	0.003 (0.002)
Sitagliptin	5.25 (11.6)	11.8 (4.62)	7.42 (0.99)	12.8 (29.0)	0.19 (0.02)	4.99 (0.76)	13.93 (2.13)	5.86 (1.72)	3.661 (0.404)	0.139 (0.022)	0.050 (0.008)	0.118 (0.031)
Sotalol	8.09 (4.88)	3.48 (0.28)	2.83 (0.28)	1.89 (0.10)	0.67 (0.10)	6.89 (0.41)	11.99 (1.55)	6.43 (0.24)	1.034 (0.156)	0.101 (0.006)	0.058 (0.007)	0.108 (0.004)
Sulfamethxazole	3.78 (1.79)	1.10 (0.12)	1.28 (0.05)	1.22 (0.09)	Inf (453 (874))	56.34 (13.71)	29.06 (2.15)	Inf (252 (320))	0.002 (0.002)	0.012 (0.003)	0.024 (0.002)	0.003 (0.003)
Valsartan	2.26 (0.84)	1.16 (0.14)	1.23 (0.03)	1.01 (0.01)	6.37 (0.83)	7.51 (0.27)	74.15 (8.46)	39.70 (4.38)	0.109 (0.014)	0.092 (0.003)	0.009 (0.001)	0.017 (0.002)
Venlafaxine	47.6 (2.10)	12.4 (2.71)	9.23 (1.58)	12.9 (1.34)	0.97 (0.12)	3.59 (0.33)	8.17 (1.35)	4.79 (0.32)	0.718 (0.092)	0.193 (0.018)	0.085 (0.014)	0.145 (0.010)

Table S4 Root mean square errors (RMSE) and median likelihoods of reactive model fits on Flowpaths a, b, c and d of Flume 1; Measurement precision as relative standard deviation and realted DT50 thresholds.

**Compound**	**RMSE**				**Median Likelihood**				**Measurement precision Rel. Stdv [%]**	**DT50 thresholds [h] based on the measurement precision**			
	**a**	**b**	**c**	**d**	**a**	**b**	**c**	**d**		**a**	**b**	**c**	**d**
1H-Benzotriazole	0.482	0.471	0.248	0.655	-3.07	-5.93	-1.8	-5.74	8.95	112.6	179.6	318.8	148.0
Acesulfame	1.415	1.801	1.852	0.926	-86.14	-172.63	-250.49	-109.51	1.40	746.4	1190.3	2112.6	981.1
Bezafibrate	1.258	0.696	0.844	1.049	-19.49	-10.1	-14.42	-6.42	5.81	176.5	281.5	499.5	232.0
Carbamazepine	0.945	0.513	0.421	1.478	-16.37	-7.1	-1.07	-20.17	7.94	127.5	203.4	361.0	167.6
Clofibric acid	0.724	1.279	0.966	0.649	-10.51	-22.47	-16.29	-5.69	4.85	212.6	339.0	601.7	279.4
Diclofenac	1.097	0.569	0.546	1.441	-10.19	-4.38	-16.88	-20.66	6.03	169.6	270.6	480.2	223.0
Furosemide	3.507	1.750	3.385	1.546	-29.99	-16.15	-7.26	-9.3	16.52	58.5	93.3	165.5	76.9
Gemfibrozil	0.093	0.266	0.311	0.445	2.38	-4.3	-14.11	-9.58	4.14	249.8	398.3	707.0	328.3
Hydrochlorothiazide	0.744	0.275	0.312	1.599	-21.08	-1.15	-2.29	-50.88	3.31	313.8	500.5	888.3	412.5
Ibuprofen	2.317	0.531	2.847	1.215	-6.39	-0.39	-5.36	-2.67	13.40	73.4	117.0	207.7	96.5
Irbesartan	1.071	0.924	0.337	0.945	-285.02	-201.2	-32.17	-43.31	4.62	223.2	356.0	631.9	293.4
Ketoprofen	1.628	1.301	1.867	1.616	-14.52	-5.88	-19.82	-13.71	9.87	101.6	162.0	287.6	133.6
Metformin	0.665	0.298	0.165	0.230	-31.19	8.23	-84.69	1.85	3.67	282.6	450.7	800.0	371.5
Naproxen	1.617	0.909	1.204	1.702	-15.49	-8.48	-8.88	-17.27	6.88	148.2	236.4	419.5	194.8
Sitagliptin	0.247	0.051	0.039	0.115	-16.5	9.62	10.17	4.26	22.05	42.4	67.6	120.0	55.7
Sotalol	0.058	0.081	0.066	0.082	10.5	9.39	12.26	7.76	2.18	479.7	765.0	1357.7	630.5
Sulfamethoxazole	0.750	0.241	0.699	0.734	-7.66	1.8	-14.58	-15.77	10.46	95.5	152.4	270.5	125.6
Valsartan	0.325	0.284	1.543	1.959	3.75	-24.23	-65.43	-65.47	4.71	219.0	349.2	619.8	287.8
Venlafaxine	1.504	0.059	0.857	0.060	-109	-3.52	-38.97	3.19	9.27	108.5	173.0	307.1	142.6

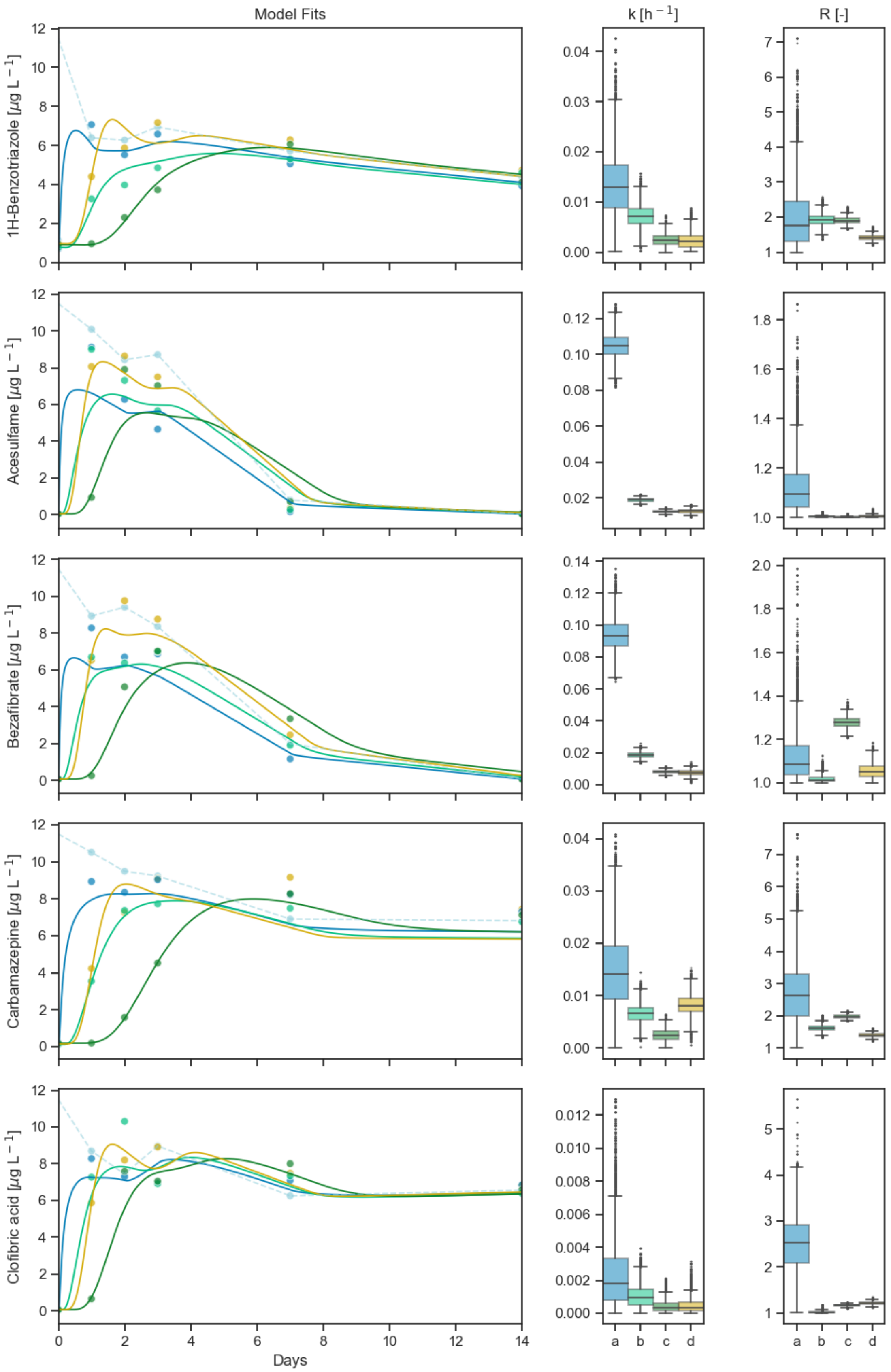

Figure S5 Measured concentrations and modeled break through curves of 1H-benzotriazole, acesulfame, bezafibrate, carbamazepine and clofibric acid in Flume 1. To the right, the estimated posterior distributions (n=40040) of the degradation rate constant *k* and the retardation coefficient *R* are given for each Flowpath a, b, c and d.

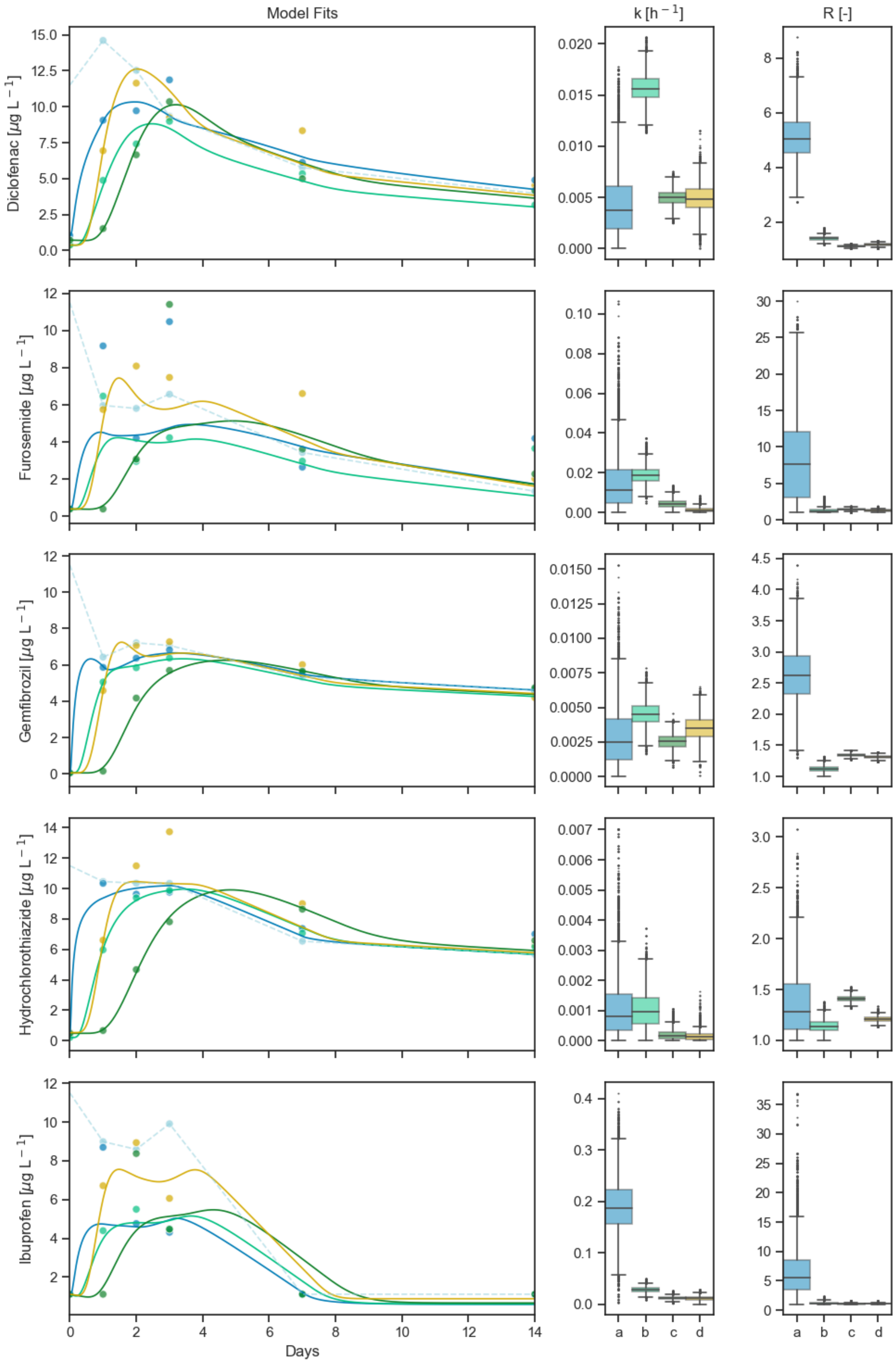

Figure S6 Measured concentrations and modeled break through curves of diclofenac, furosemide, gemfibrozil, hydrochlorothiazide and ibuprofen in Flume 1. To the right, the estimated posterior distributions (n=40040) of the degradation rate constant *k* and the retardation coefficient *R* are given for each Flowpath a, b, c and d.

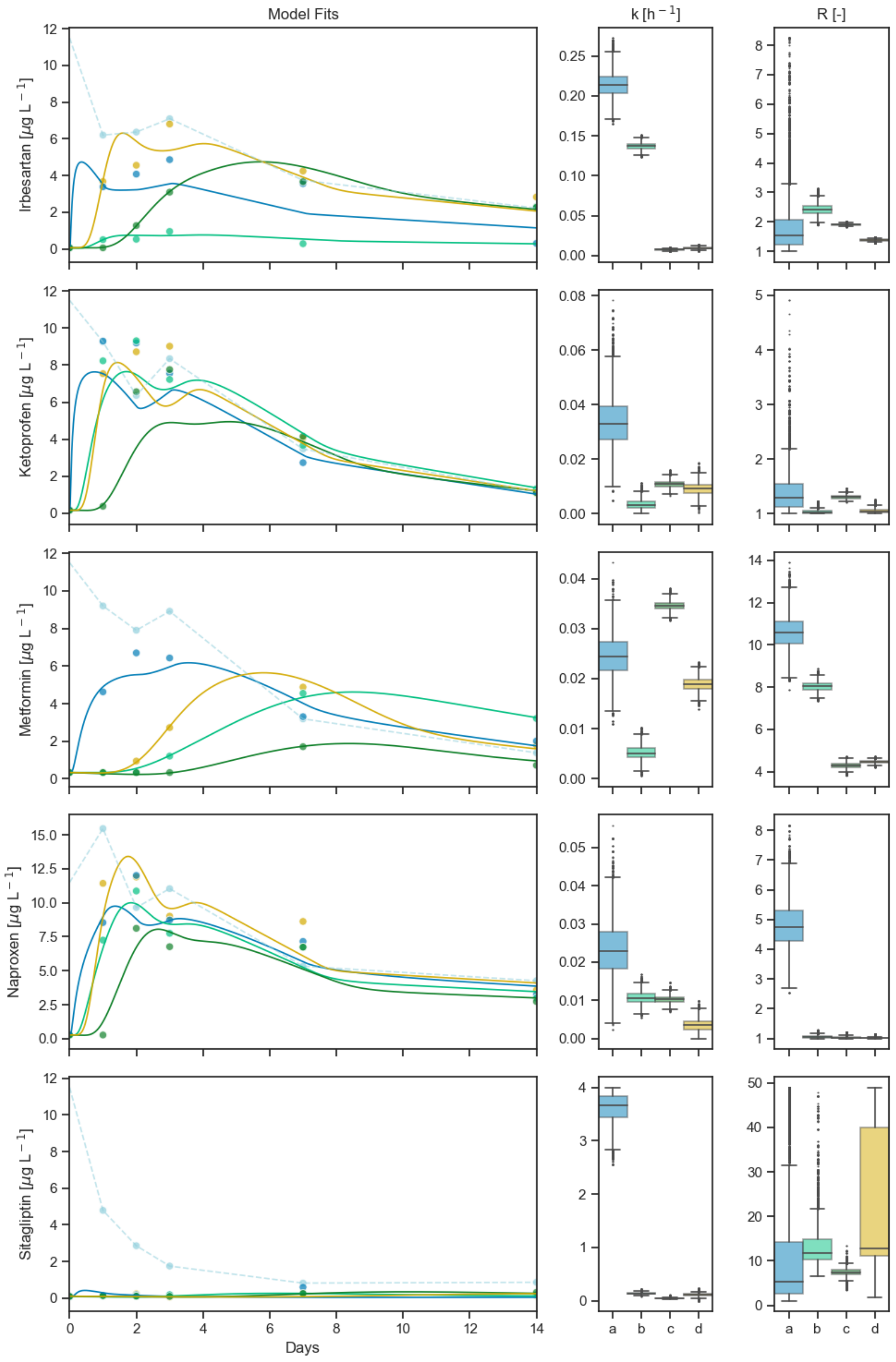

Figure S7 Measured concentrations and modeled break through curves of irbesartan, ketoprofen, metformin, naproxen and sitagliptin in Flume 1. To the right, the estimated posterior distributions (n=40040) of the degradation rate constant *k* and the retardation coefficient *R* are given for each Flowpath a, b, c and d.

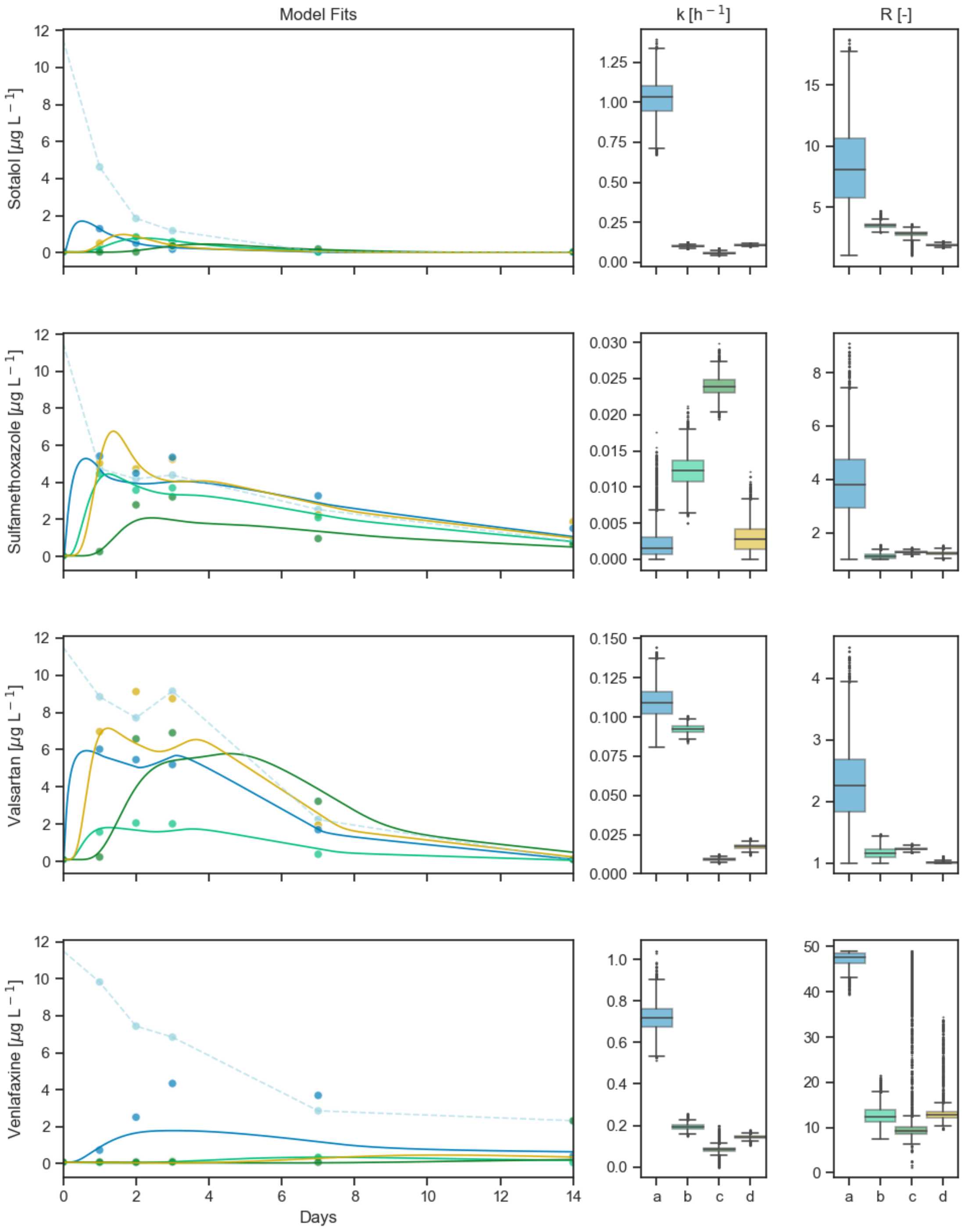

Figure S8 Measured concentrations and modeled break through curves of sotalol, sulfamethoxazole, valsartan and venlafaxine in Flume 1. To the right, the estimated posterior distributions (n=40040) of the degradation rate constant *k* and the retardation coefficient *R* are given for each Flowpath a, b, c and d.

Sorption, retardation or biodegradation?

All parent compounds were injected at day 0 at the same time in the same concentrations. So what caused the differences observed in concentration curves in the sediment (Figure 2)? A major explanation is the individual susceptibility of the compounds to external conditions and to different processes. The main processes affecting the compounds in the sediment are biodegradation and sorption. Biodegradation is a microbial breakdown of the compound molecules either into transformation products (transformation) or a complete breakdown into its inorganic parts (mineralisation). The two processes are not distinguishable by the compound concentration, but transformation can be identified by analysis of transformation products. Either way, biodegradation reduces the concentration of the parent compound. Potential reversibility of biodegradation is mostly disregarded, but it is conceivable for instance in the case of sulfamethoxazole [3]. Sorption, in turn, can occur on a scale from quickly reversible to completely irreversible. Irreversible sorption to clay particles or organic matter reduces the concentrations measured in the PW. Quickly reversible sorption increases the travel time of a compound in the sediment and is also referred to as retardation, but does not affect the overall concentration. Irreversible sorption is assumed to be of little importance in the flume sediments due to a low carbon content on the one side and a low content of fine sediment material on the other side, both major binding sites for organic compounds. The expectation was confirmed by sorption tests of carbamazepine, ibuprofen and sulfamethoxazole yielding no sorption and by a generally low cation exchange capacity of the sediment [4]. However, retardation of compounds in the sediment is expected, although to lower extent as observed in sediments of high carbon content and fine material as in River Erpe. [5]

References

1 Posselt, M., Jaeger, A., Schaper, J. L., Radke, M. & Benskin, J. P. Determination of polar organic micropollutants in surface and pore water by high-resolution sampling-direct injection-ultra high performance liquid chromatography-tandem mass spectrometry. *Environmental Science: Processes & Impacts* **20**, 1716-1727, doi:10.1039/C8EM00390D (2018).

2 Posselt, M. *et al.* Bacterial Diversity Controls Transformation of Wastewater-Derived Organic Contaminants in River-Simulating Flumes. *Environ. Sci. Technol.* **54**, 5467-5479, doi:10.1021/acs.est.9b06928 (2020).

3 Radke, M., Lauwigi, C., Heinkele, G., Murdter, T. E. & Letzel, M. Fate of the antibiotic sulfamethoxazole and its two major human metabolites in a water sediment test. *Environ Sci Technol* **43**, 3135-3141 (2009).

4 Jaeger, A. *et al.* Using recirculating flumes and a response surface model to investigate the role of hyporheic exchange and bacterial diversity on micropollutant half-lives. *Environmental Science: Processes & Impacts* **21**, 2093-2108, doi:10.1039/C9EM00327D (2019).

5 Schaper, J. L. *et al.* Fate of Trace Organic Compounds in the Hyporheic Zone: Influence of Retardation, the Benthic Biolayer, and Organic Carbon. *Environ. Sci. Technol.* **53**, 4224-4234, doi:10.1021/acs.est.8b06231 (2019).
